# Pleiotropic effects of *PAB1* deletion: Extensive changes in the yeast proteome, transcriptome, and translatome

**DOI:** 10.1371/journal.pgen.1011392

**Published:** 2024-09-05

**Authors:** Kotchaphorn Mangkalaphiban, Robin Ganesan, Allan Jacobson

**Affiliations:** Department of Microbiology, UMass Chan Medical School, Worcester, Massachusetts, United States of America; Ohio State University, UNITED STATES OF AMERICA

## Abstract

Cytoplasmic poly(A)-binding protein (PABPC; Pab1 in yeast) is thought to be involved in multiple steps of post-transcriptional control, including translation initiation, translation termination, and mRNA decay. To understand both the direct and indirect roles of PABPC in more detail, we have employed mass spectrometry to assess the abundance of the components of the yeast proteome, as well as RNA-Seq and Ribo-Seq to analyze changes in the abundance and translation of the yeast transcriptome, in cells lacking the *PAB1* gene. We find that *pab1Δ* cells manifest drastic changes in the proteome and transcriptome, as well as defects in translation initiation and termination. Defects in translation initiation and the stabilization of specific classes of mRNAs in *pab1Δ* cells appear to be partly indirect consequences of reduced levels of specific initiation factors, decapping activators, and components of the deadenylation complex in addition to the general loss of Pab1’s direct role in these processes. Cells devoid of Pab1 also manifested a nonsense codon readthrough phenotype indicative of a defect in translation termination. Collectively, our results indicate that, unlike the loss of simpler regulatory proteins, elimination of cellular Pab1 is profoundly pleiotropic and disruptive to numerous aspects of post-transcriptional regulation.

## Author summary

Many human diseases are caused by having too much or too little of certain cellular proteins. The amount of an individual protein is influenced by the level of its messenger mRNA (mRNA) and the efficiency of translation of the mRNA into a polypeptide chain by the ribosomes. Cytoplasmic poly(A)-binding protein (PABPC) plays numerous roles in the regulation of this multi-staged process, but understanding its specific role has been challenging because it is sometimes unclear whether experimental results are related to PABPC’s direct role in a specific biochemical process or to indirect effects of its other roles, leading to conflicting models of PABPC’s functions between studies. Here, we characterized defects in different stages of protein synthesis in response to loss of PABPC in yeast cells by measuring whole-cell levels of mRNAs, ribosome-associated mRNAs, and proteins. We demonstrated that defects in most steps of protein synthesis other than the last can be explained by reduced levels of mRNAs that code for proteins important for that step in addition to loss of PABPC’s direct role on that step. Our data and analyses serve as resources aiding interpretation of experiments in which PABPC levels have been manipulated and generally point to the resulting complexity of such manipulations.

## Introduction

Eukaryotic mRNAs are subject to complex post-transcriptional regulation by RNA binding proteins (RBPs) that control protein output. Cytoplasmic poly(A)-binding proteins (PABPCs) are RBPs that bind polyadenylated tails at mRNA 3’-ends and subsequently play roles in multiple stages of cytoplasmic mRNA regulation, from translation initiation to termination and mRNA decay [1–3]. The numerous functions of PABPC are attributed to its ability to interact with not only mRNAs but also various other proteins to form messenger ribonucleoprotein (mRNP) complexes [2]. PABPC’s conserved structure consists of three regions: i) four RNA recognition motif (RRM) domains, two of which (RRM1 and RRM2) bind 12 adenosines with high affinity [3,4], while the protein overall covers 27 adenosines [5], ii) a proline-rich (P) linker domain, and iii) a C-terminal mademoiselle (MLLE) domain that interacts with other proteins via their PABP-interacting motif 2 (PAM2) [2,3,6]. Mammals have multiple isoforms of PABPCs, of which the most ubiquitous isoform as well as the most studied is PABPC1, whereas the yeast *Saccharomyces cerevisiae* has only one PABPC, Pab1 [2,3,6,7].

The current model for general mRNA decay in yeast involves biphasic poly(A) tail shortening by Pan2-Pan3 and Ccr4-Not deadenylase complexes, decapping by the Dcp1/Dcp2 holoenzyme, and exonucleolytic Xrn1-mediated 5’-3’ degradation or exosome-mediated 3’-5’ degradation [3,8,9]. PABPC appears to have a paradoxical role in these processes. On the one hand, PABPC1/Pab1 stimulates deadenylation by recruiting Pan2-Pan3 to the poly(A) tail through its interaction with the PAM2 motif on Pan3 [1,3,8,10,11]. Consistent with this model, mRNAs in yeast cells harboring a deletion of *PAB1* or mutations of Pab1’s C-terminal Pan3-interacting domain had longer poly(A) tails than their counterparts in wild-type cells [1–3,12–14]. On the other hand, PABPC has been shown to protect mRNAs from exonucleases [3,8].

Importantly, mRNAs with longer poly(A) tails are generally more stable and better translated than their short-tailed or un-tailed counterparts [3,15–23], observations that led us to propose a role for PABPC in the enhancement of translation initiation by the possible formation of an mRNA “closed-loop” [23–25]. Current elaborations of this model postulate that mRNAs could be circularized by a chain of interactions between poly(A)-associated PABPC and 5’ end-localized initiation factors, giving rise to a poly(A) tail-PABPC-eIF4G-eIF4E-5’cap network [2,26–30]. eIF4G has been shown to interact with PABPC’s RRM2 domain [26,31–33], and disrupting this interaction reduced translation [6,29,34]. Thus, PABPC can stabilize the cap-binding complex and aid the recruitment of the 43S pre-initiation complex to the mRNA [2,3,35]. However, this closed-loop arrangement is not required for all mRNAs or all conditions, raising the question of how else 5’-3’ communication is facilitated or whether it is indeed a universal step [30,36–39]. Depletion of PABPC1 in mammalian cells had minimal effects on transcriptome-wide translation efficiency [40,41], suggesting that the stimulatory effect of PABPC on translation initiation may be restricted to circumstances where translation initiation efficiency is rate-limited.

In addition to its interactions with initiation factors, PABPC also interacts with the release factor eRF3 via its PAM2 motif in metazoans and its P-C domains in yeast [2,6,42–45]. Translation termination involves stop codon recognition in the ribosomal A-site and nascent peptide release by eRF1, whose hydrolysis function and conformational change are stimulated by eRF3’s GTPase activity [46,47]. PABPC is thought to enhance termination efficiency by promoting the recruitment of the eRF1-eRF3 release factor complex to the stop codon. The role of PABPC in termination is also inferred from: i) the observation that tethering PABPC1 or Pab1 downstream of premature termination codons (PTCs) antagonized nonsense-mediated mRNA decay (NMD), an mRNA decay pathway thought to be activated by the reduced termination efficiency of premature translation termination [48,49] and ii) the increased termination efficiency observed with proximity of a stop codon to the mRNA 3’ end [50,51]. Direct evidence for PABPC’s ability to enhance termination includes: i) addition of PABPC1 to an *in vitro* termination assay improved termination efficiency [52], ii) PABP-interacting protein PAIP1 and PAIP2 competed with eRF3 for free PABPC binding, reducing termination efficiency of PTCs *in vitro* [53], and iii) deletion of *PAB1* or Pab1’s P-C domains *in vivo* increased stop codon readthrough efficiency of reporter PTCs in a proximity-dependent manner [51]. However, as with initiation, a full understanding of PABPC’s role during termination of endogenous mRNAs *in vivo* is still lacking.

PABPC’s involvement in many major stages of mRNA regulation and translation complicate attempts to define a specific role for PABPC *in vivo* by deleting, depleting, overexpressing, or mutating the protein and have led to conflicting models of PABPC’s function. Therefore, to specifically assess direct and indirect consequences of deleting PABPC, we generated and analyzed mass spectrometry, RNA-Seq, and ribosome profiling [54] data from yeast cells lacking Pab1. As expected, protein and mRNA abundance changed substantially in *pab1Δ* cells. We found that deleting *PAB1* resulted in a translation termination defect that appears to be at least partially due to reduced eRF3 protein level as well as to loss of Pab1’s stimulatory function on termination. In addition, translation initiation defects and changes in relative translation efficiency in *pab1Δ* cells may be confounded by reduced initiation factor levels, especially eIF4G and eIF1. Further, an analysis of decapping activator substrates revealed that increased levels of certain mRNA subgroups may be partially caused by reduced levels of a specific decapping activator or components of Ccr4-Not deadenylation complex. Together, our results catalog the consequences of *PAB1* deletion and illustrate the complexity of Pab1’s pleiotropic effects on the transcriptome-wide regulation of translation and mRNA decay.

## Results

### Deletion of *PAB1* results in significant changes in the yeast proteome and transcriptome

**Fig 1:**
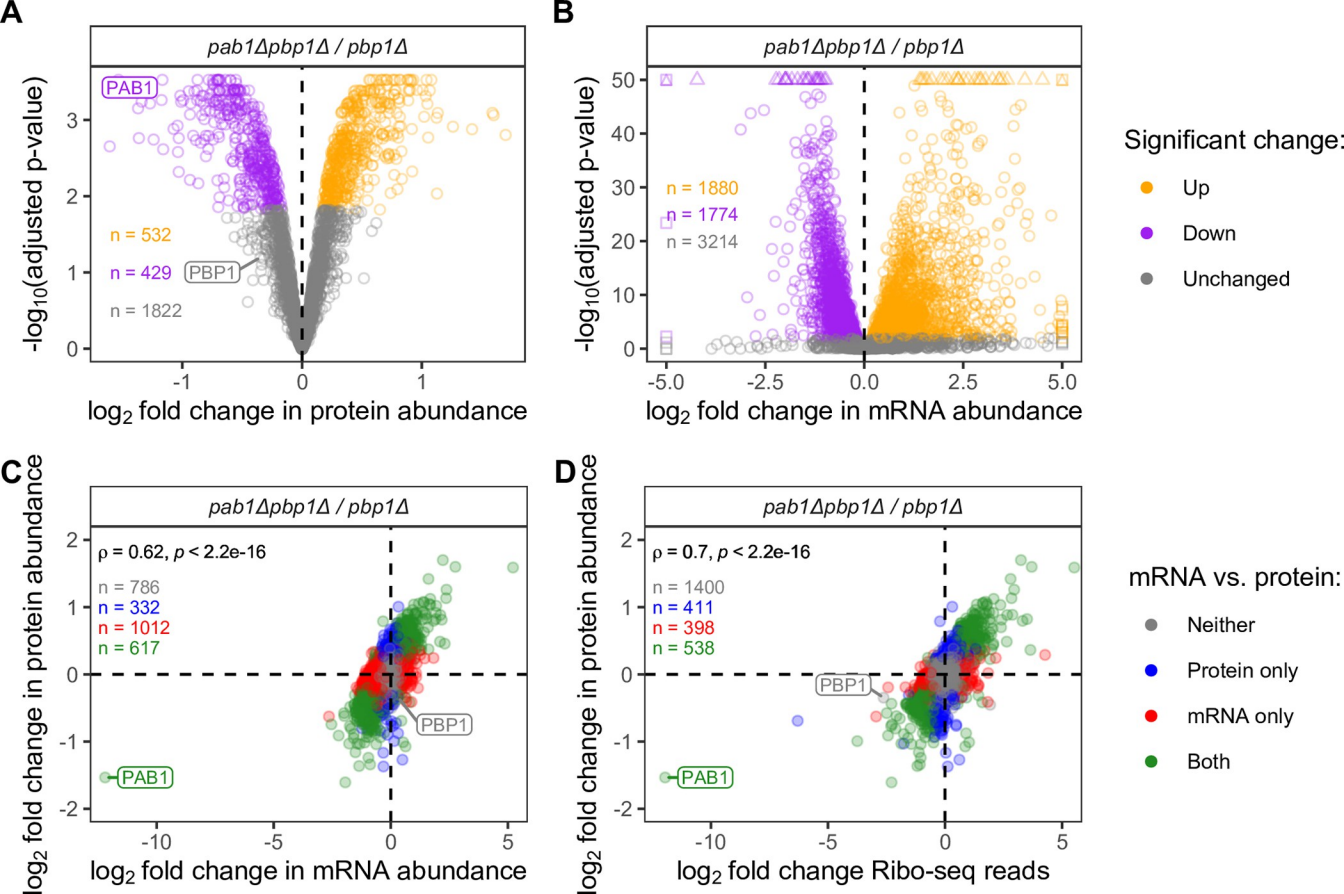
*PAB1* gene deletion has significant effects on the transcriptome and the proteome. **A.** Volcano plot of changes in proteome (mass spectrometry data) between *pab1Δpbp1Δ* and *pbp1Δ* strains. Orange, purple, and grey dots represent proteins with higher abundance (positive log_2_ fold change, adjusted p-value < 0.015), lower abundance (negative log_2_ fold change, adjusted p-value < 0.015), and no change (adjusted p-value ≥ 0.015), respectively, in the *pab1Δpbp1Δ* strain. **B.** Volcano plot of changes in transcriptome (RNA-Seq data) between *pab1Δpbp1Δ* and *pbp1Δ* strains. Orange, purple, and grey dots represent mRNAs with higher abundance (positive log_2_ fold change, adjusted p-value < 0.01), lower abundance (negative log_2_ fold change, adjusted p-value < 0.01), and no change (adjusted p-value ≥ 0.01), respectively, in the *pab1Δpbp1Δ* strain. **C.** Comparison of log_2_ fold change in transcriptome (RNA-Seq reads) and proteome (mass spectrometry quantification), with Spearman’s correlation coefficient. **D.** Comparison of log_2_ fold change in ribosome profiling (Ribo-Seq) reads and proteome (mass spectrometry quantification), with Spearman’s correlation coefficient. For C and D: Grey, genes whose mRNA and protein abundance remained unchanged. Blue, genes whose protein but not mRNA abundance changed significantly. Red, genes whose mRNA but not protein abundance changed significantly. Green, genes whose mRNA and protein abundance both changed significantly.

**Fig 2:**
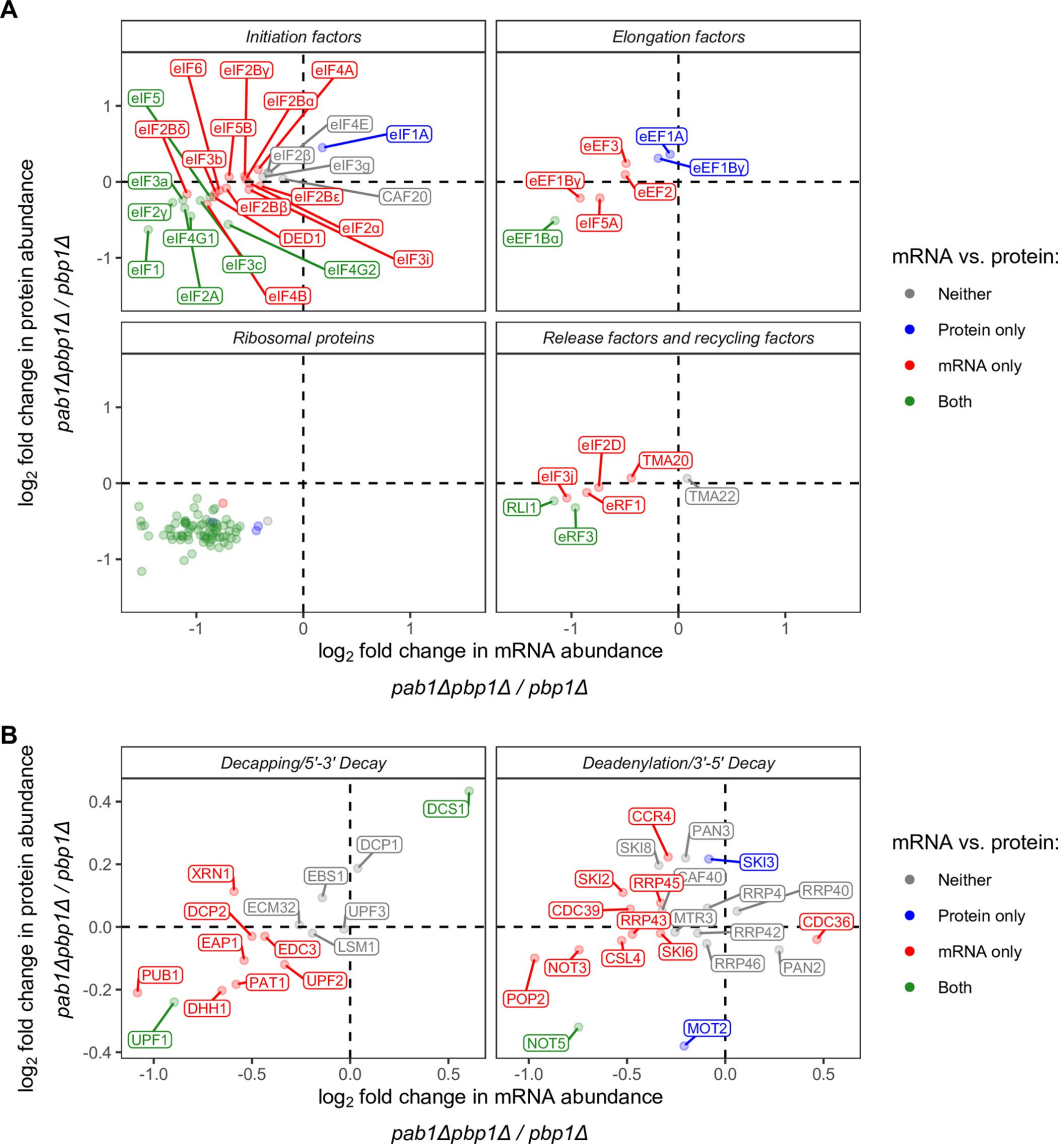
*PAB1* gene deletion significantly decrease mRNA and protein levels of many translation- and RNA binding-related genes. Data as in Fig 1C, with the focus on translation-related genes (**A**) or mRNA decay-related genes (**B**). The ribosomal proteins depicted here account for 94% of all ribosomal proteins that make up the 40S and 60S subunits.

**Fig 3:**
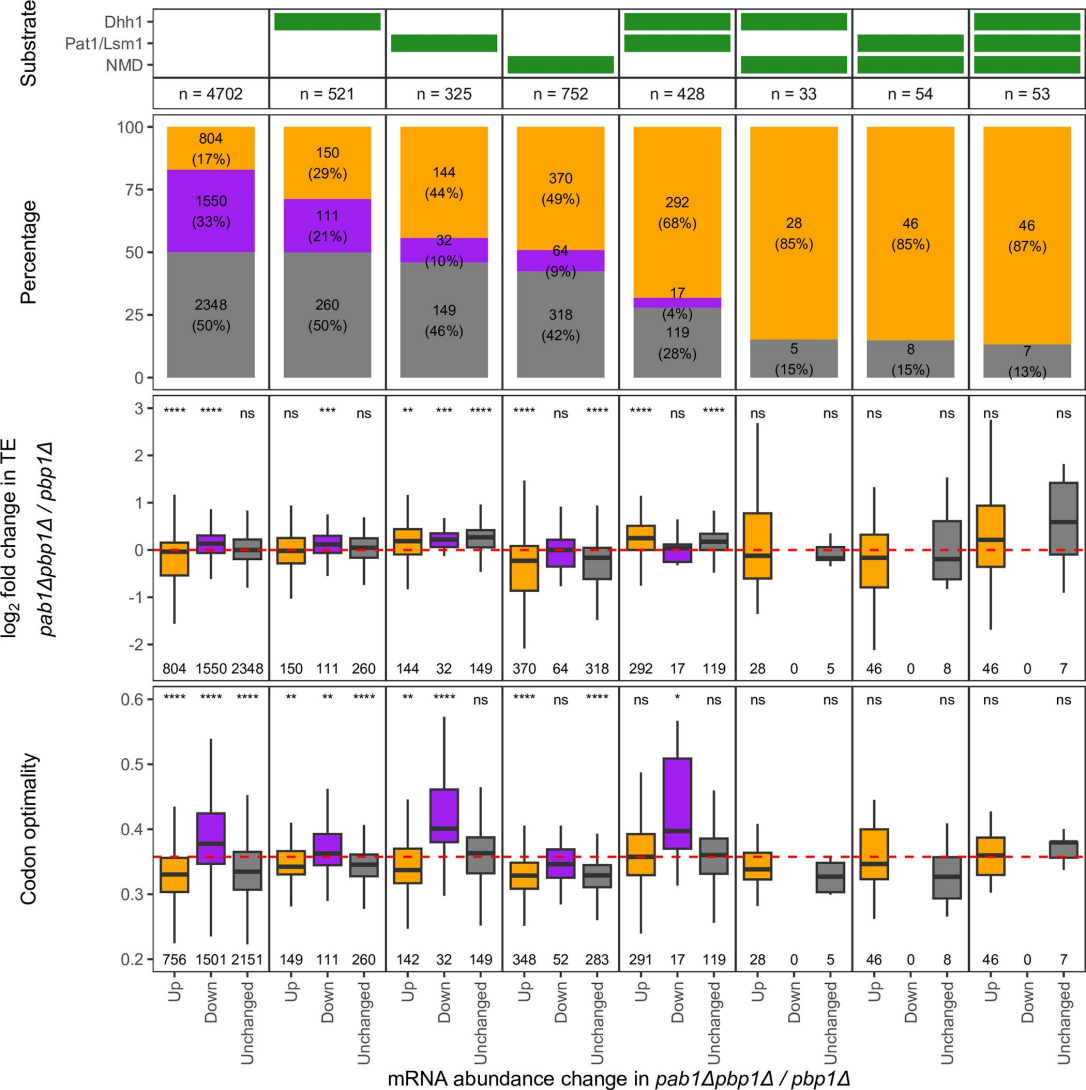
Changes in the abundance of decapping activator substrates by different consequences of *PAB1* deletion. mRNA abundance and translation efficiency changes between *pab1Δpbp1Δ* and *pbp1Δ* strains for Dhh1, Pat/Lsm1, and NMD substrates. ***(Row 1)*** Panel indicating substrate status (green) of panels below. A substrate is defined as an mRNA that is upregulated upon decapping deactivator gene deletion [9,59]. Dhh1: mRNAs upregulated in a *dhh1Δ* strain relative to WT [59]; Pat1/Lsm1: mRNAs commonly upregulated in *pat1Δ* and *lsm1Δ* strains relative to WT [59]; NMD: mRNAs commonly upregulated in *upf1Δ*, *upf2Δ*, and *upf3Δ* strains relative to WT [58]. ***(Row 2)*** Proportions of mRNA abundance changes from Fig 1B: Up (orange), Down (purple), and Unchanged (grey), separated into columns by substrate status. ***(Row 3)*** Distribution of log_2_ fold change in TE between *pab1Δpbp1Δ* and *pbp1Δ* strains for mRNA groups from Row 2 panel. Two-sided Wilcoxon’s rank sum test with Benjamini-Hochberg method for multiple-testing correction was used to compare median log_2_ fold change in TE to zero (no change, red dashed line). ***(Row 4)*** Distribution of codon optimality score for mRNA groups from Row 2 panel. Two-sided Wilcoxon’s rank sum test with Benjamini-Hochberg method for multiple-testing correction was used to compare median codon optimality score in each group to the sample’s mean score (red dashed line). Significant levels were reported as the following: (ns) not significant, (*) p < 0.05, (**) p < 0.01, (***) p < 0.001, (****) p < 0.0001.

To investigate proteome- and transcriptome-wide abundance changes when Pab1 is absent, we performed mass spectrometry, RNA-Seq, and ribosome profiling analyses of yeast cells harboring a *PAB1* deletion. Because *PAB1* is an essential gene, the deletion was created in a *pbp1Δ* background, which suppresses *pab1Δ* lethality [55], and we used the *PAB1/pbp1Δ* strain as our wild-type *PAB1* control. Proteomic and transcriptomic data obtained from three biological replicates of each strain were reproducible, as evidenced by high Pearson’s correlation coefficients between replicates (S1 Fig). Differential expression analyses were performed for mass spectrometry and RNA-Seq data for each pair of yeast strains to assess relative changes in the abundance of specific proteins and mRNAs (Figs 1–3 and S2). For proteins that were detectable by mass spectrometry, relative changes in their mRNA and protein abundance are quite consistent with each other, with Spearman’s correlation of 0.62–0.74 (Figs 1C and S2C). Similarly, relative changes in ribosome profiling reads are also consistent with protein abundance changes, with Spearman’s correlation of 0.6–0.72 (Figs 1D and S2D).

Pbp1 is a Pab1-interacting protein that has been implicated in polyadenylation, cell growth in non-fermentable carbon source, and mitochondrial biogenesis [55–57]. Consistent with previous findings, deletion of *PBP1* has a minimal impact on mRNA and protein abundance relative to isogenic wild-type (WT) cells (Figs S1 and S2A left panel, and S2B left panel) grown in YEPD media. Additionally, gene ontology analyses revealed that proteins that were down-regulated are associated with mitochondrial-related pathways (S1 Table and S3 Fig). On the other hand, as expected of Pab1’s essential and extensive role in mRNA stability regulation, relative protein and mRNA levels changed drastically when Pab1 was absent (Figs 1A, 1B, S2A right panel, and S2B right panel). Gene ontology analyses showed that up-regulated proteins belong to metabolic pathways, while down-regulated proteins are related to ribosome biogenesis, translation, and RNA-binding (S1 Table and S4 Fig). Indeed, the *pab1Δ* mutation led to substantial reduction in the levels of ribosomal proteins (Fig 2A, bottom left), multiple initiation factors (Fig 2A, top left), and some mRNA decay factors (Fig 2B). These results raised the question of whether the roles of Pab1 in translation and mRNA stability borne out of Pab1 perturbation experiments are at least partially attributable to this reduction in key proteins in these pathways in addition to Pab1’s direct roles in these processes. Hence, we explored the phenotypes of mRNA decay, translation initiation, translation efficiency, and translation termination upon *PAB1* deletion, focusing on analyses of *pab1Δpbp1Δ* cells relative to *pbp1Δ* cells, and then considered whether the observed changes were direct or indirect effects of *PAB1* deletion.

### Substrates of decapping activators Pat1/Lsm1 and Upf1/Upf2/Upf3 tend to be more increased than decreased in response to *PAB1* deletion

Among its many functions, PABPC has important regulatory roles in mRNA decay [1–3]. Hence, we asked how the absence of Pab1 impacts the levels of mRNAs that are substrates of different decapping activators, namely Dhh1, Pat1/Lsm1, and the Upf factors of the NMD pathway. Dhh1, Pat1/Lsm1, and NMD substrates are defined respectively as mRNAs whose levels were increased in *dhh1Δ* cells, commonly increased in *pat1Δ* and *lsm1Δ* cells, and commonly increased in *upf1Δ*, *upf2Δ*, *and upf3Δ* cells, relative to WT [58,59]. Approximately half of the mRNAs in each group showed significant changes in abundance in *pab1Δpbp1Δ* relative to *pbp1Δ* cells (Fig 3). Of mRNAs that showed significant changes, those that are targets of either Pat1/Lsm1 or NMD or both tend to be increased rather than decreased (Fig 3, row 2, columns 3–8, compare Up (orange) to Down (purple)). This trend does not apply to the Dhh1-only substrates, however, since there seem to be comparable proportions of Dhh1-only substrates that are increased vs. decreased (29% vs. 21%) (Fig 3, row 2, column 2, compare Up (orange) to Down (purple)).

To determine whether increases in the abundance of Pat1/Lsm1 and Dhh1 substrates, which follow the canonical deadenylation-dependent pathway, were due to loss of Pab1’s role in deadenylase recruitment or dysregulation of decay pathway components, we investigated changes in mRNA and protein levels of genes involved in decapping, deadenylation, and decay (Fig 2B). We found that while decapping enzyme subunits (Dcp1 and Dcp2), Pat1, Lsm1, Dhh1, the exonuclease Xrn1, and exosome components are not significantly enriched or depleted at the protein level, two proteins that are components of the Ccr4-Not deadenylase complex, Mot2 (also commonly known as Not4) and Not5, are significantly depleted to ~77–80% of the amount in *pbp1Δ* cells (Fig 2B). Not4 and Not5 are thought to link slow translation elongation of non-optimal codons to deadenylation as well as deadenylation to decapping [3]. Specifically, Dhh1’s association with the ribosome requires Not5’s ribosome binding and Not4’s E3 ligase activity to ubiquitinate 40S ribosomal subunit protein eS7 [60]. Not5 has also been shown to bind Pat1 to promote decapping [61]. Thus, stabilization of Pat1/Lsm1 and some Dhh1 substrates may be partially attributable to dysregulated Ccr4-Not components, in addition to loss of Pab1’s role in deadenylase recruitment.

Because decapping of NMD substrates is usually a deadenylation-independent mechanism that is triggered by premature translation termination [9,62,63], stabilization of NMD substrates in *pab1Δpbp1Δ* cells is likely unrelated to Pab1’s role in protecting the poly(A) tail or deadenylase recruitment. Rather, it is likely due to decreases in translation, which lead to decreased frequency of premature termination. To test whether NMD substrates have a decreased initiation rate in the absence of Pab1, we calculated translation efficiency (TE) of each mRNA in each yeast strain by normalizing ribosome profiling reads in the protein-coding (CDS) region to mRNA level and compared relative log_2_ fold change in TE between *pab1Δpbp1Δ* and *pbp1Δ* strains for each group of mRNAs to the value of unchanged TE (log_2_ fold change = 0) (Fig 3, row 3). Consistent with our hypothesis, we found that NMD substrates that are significantly stabilized or have unchanged mRNA levels in *pab1Δpbp1Δ* have relatively lower TE while the minority that are depleted have relatively unchanged TE (Fig 3, row 3, column 4). However, reduced TE is probably not the only contribution to NMD substrate enrichment, as the protein level of the key NMD protein Upf1 is significantly reduced in *pab1Δpbp1Δ* cells to ~85% of the level in *pbp1Δ* cells (Fig 2B).

Since rapid mRNA decay can be triggered by slow translation elongation while mRNAs can evade deadenylation when they have optimal codons and are efficiently translated [64,65,3], we wondered whether mRNAs otherwise stabilized in WT cells would be destabilized in the absence of Pab1. We investigated whether mRNAs down-regulated in *pab1Δpbp1Δ* cells, such as mRNAs of ribosomal proteins, are such “optimal transcripts” by calculating a codon optimality score for each transcript (Fig 3, row 4). Except for NMD substrates which are known to be non-optimal [58], Dhh1 and Pat1/Lsm1 substrates, and non-substrates in the Down group all have higher codon optimality scores than the sample average (Fig 3, row 4), suggesting that they are more sensitive to *PAB1* deletion.

Overall, we demonstrated that targets of mRNA decay pathways followed the expected phenotypes upon *PAB1* deletion, namely the tendency to stabilize, suggesting direct role of Pab1 in mRNA decay regulation. However, the depletion of some factors in the pathways upon *PAB1* deletion cannot be completely ruled out as an indirect effect of this stabilization.

### Deletion of *PAB1* leads to translation initiation defects but has minimal effects on translation efficiency

**Fig 4:**
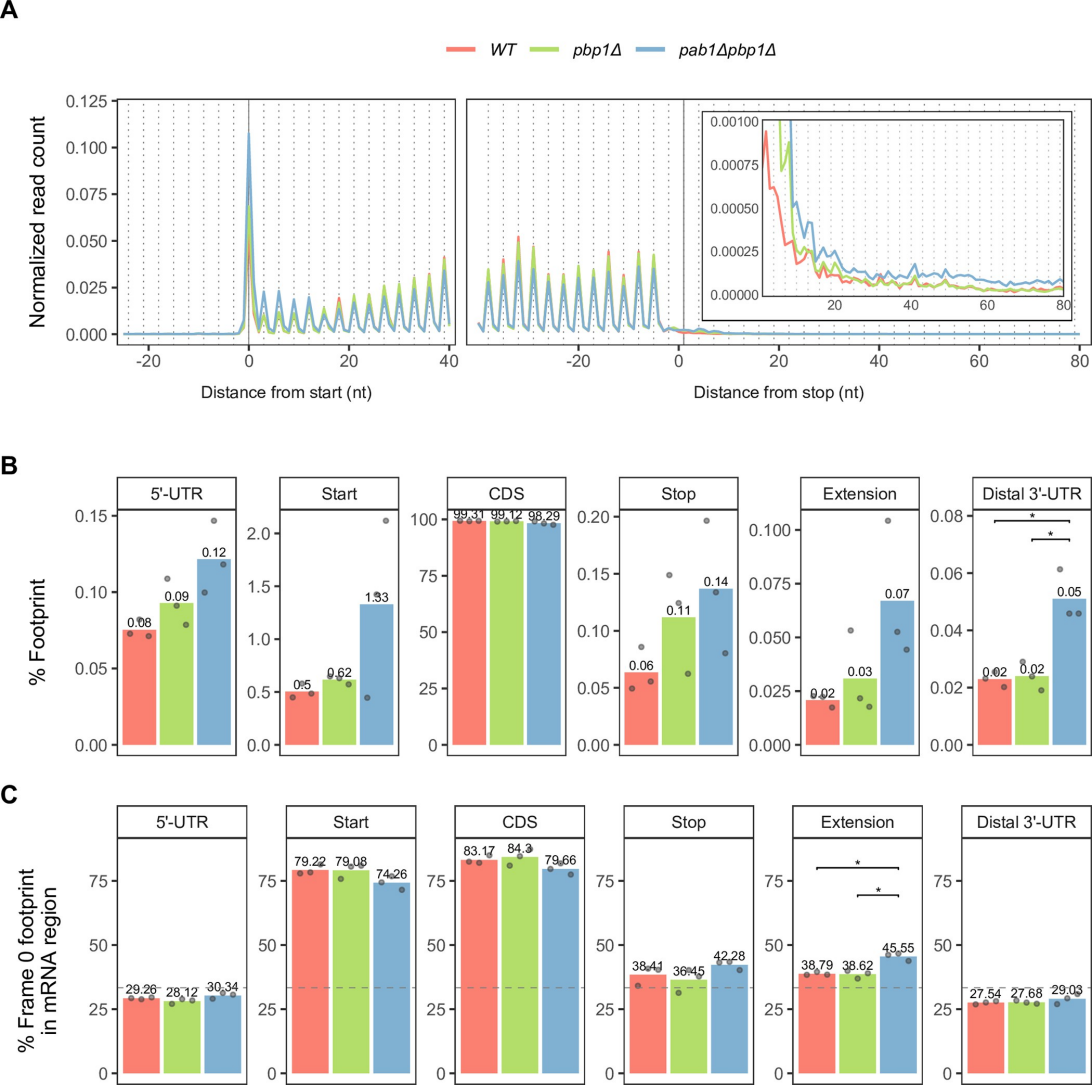
*PAB1* gene deletion leads to ribosome accumulation at mRNA start codons and in UTR regions. **A.** Ribosome footprints (replicate libraries were pooled) were counted by their P-site positions in the indicated nucleotide window around the canonical CDS’s start and stop coordinates (the first nucleotide of AUG and the last nucleotide of the last amino acid-encoding codon) of annotated ORFs. Raw footprint counts were normalized by the total footprint count in the windows. Inset: Magnified view of the 3’-UTR region (Distance from stop > 0 nt). **B-C.** Percentage of footprints in sequencing library belonging in different mRNA regions (**B**) and percentage of frame 0 footprints in each mRNA region, where grey dashed line indicates a theoretical 33% at which all 3 reading frames are equally represented (**C**). “Start” region includes the canonical AUG and 3 flanking nucleotides on each side. “Stop” region includes the canonical stop codon and 3 flanking nucleotides on each side. “Extension” is the region following the “Stop” until (but not including) the first in-frame stop codon in the 3’-UTR. “Distal 3’-UTR” is the 3’-UTR region following “Extension.” Percentages from individual replicate libraries (grey points) were averaged (bar plot and reported value above it). Unpaired Student’s t-test with Benjamini-Hochberg method for multiple-testing correction was used to compare values between all possible pairwise strains. Only significant comparisons were reported as the following: (*) p < 0.05, (**) p < 0.01, (***) p < 0.001, (****) p < 0.0001.

Pab1 is thought to promote translation initiation by aiding the association of the 40S ribosomal subunit and the eIF4F complex with the mRNA 5’ cap through a direct interaction with eIF4G [2,26,29,30,66,67]. Translation initiation defects may thus be expected when *PAB1* is deleted. To investigate translation initiation, we analyzed ribosome profiling data of WT, *pbp1Δ*, and *pab1Δpbp1Δ* cells. Consistent with the expectation of translating ribosomes, ribosome footprints in all strains were found mostly with their P-site locations in the coding region (CDS), showed 3-nt periodicity, and consisted predominantly of footprints in the main reading frame (frame 0) (Fig 4). However, we observed subtle increased accumulation of ribosomes at the canonical AUG start codon and in the 5’-UTR region in the *pab1Δpbp1Δ* strain (Fig 4B), indicating a possible translation initiation defect in the absence of Pab1.

**Fig 5:**
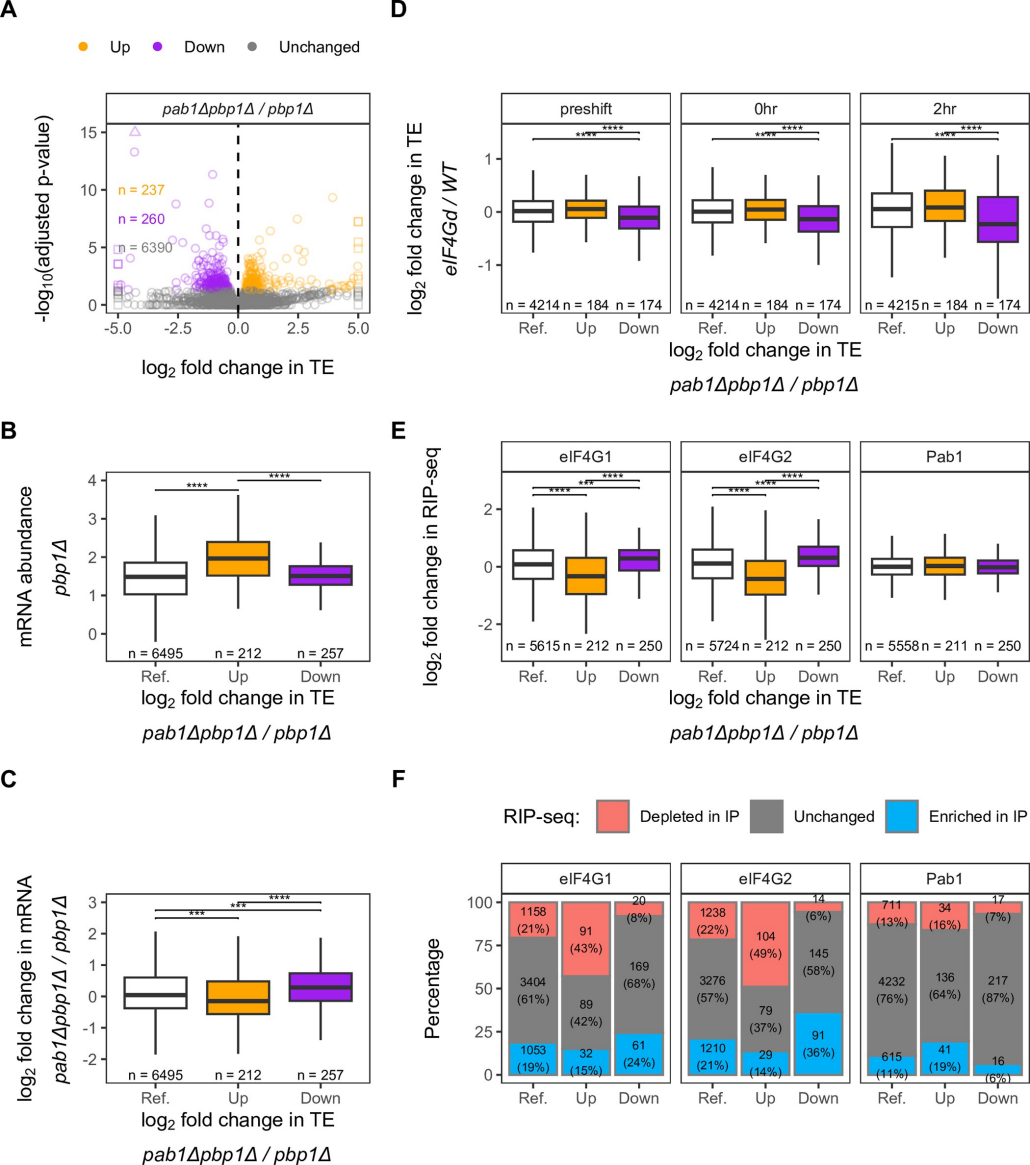
Translation initiation defects in response to *PAB1* deletion may be related to reduction in eIF4G level. **A.** Volcano plot of changes in relative translation efficiency (TE) between *pab1Δpbp1Δ* and *pbp1Δ* strains. Orange, purple, and grey dots represent mRNAs with increased (positive log_2_ fold change, adjusted p-value < 0.05), decreased (negative log_2_ fold change, adjusted p-value < 0.05), and unchanged TE (adjusted p-value ≥ 0.05), respectively, in the *pab1Δpbp1Δ* strain. **B-E.** Distribution of the *pbp1Δ* strain’s mRNA abundance, log_10_ (RPKM) from RNA-Seq data (**B**), mRNA abundance changes between *pab1Δpbp1Δ* and *pbp1Δ* cells (**C**), TE changes between eIF4G depleted (eIF4Gd) cells and isogenic WT cells [68] (**D**), and fold enrichment in eIF4G or Pab1 RIP-seq [36] (**E**) in each mRNA TE group from A. Two-sided Wilcoxon’s rank sum test with Benjamini-Hochberg method for multiple-testing correction was used to compare values between pairwise groups. Only significant comparisons were reported as the following: (*) p < 0.05, (**) p < 0.01, (***) p < 0.001, (****) p < 0.0001. **F.** Proportion and number of mRNAs from groups in A that were enriched (positive log_2_ fold change, FDR < 0.05), depleted (negative log_2_ fold change, FDR < 0.05), or unchanged (FDR ≥ 0.05) in RIP-seq data [36]. Pairwise χ^2^ test with Benjamini-Hochberg method for multiple-testing correction was used to compare between Reference, Up, and Down groups. p < 0.05 in all pairwise comparisons (exact values provided in S3 Table). For B-F, Reference (Ref.) group includes all mRNAs regardless of TE changes (Up + Down + Unchanged) to recapitulate the general distribution of measured values in the transcriptome and only spliced mRNA entries were considered.

To determine whether deletion of *PAB1* affected initiation rates of all mRNAs equally, we assessed the change in relative translation efficiency (TE) of each mRNA between *pab1Δpbp1Δ* and *pbp1Δ* strains (Fig 5A). Although ~500 mRNAs show substantive increases (“Up”) or decreases (“Down”) in relative TE, most mRNAs (>90% of the transcriptome) do not show significant changes in relative TE (Fig 5A, grey). The mostly unchanged relative TE is in line with the observation that the correlation between ribosome profiling read changes and protein abundance changes is only slightly better than that between RNA-Seq read (mRNA abundance) changes and protein abundance changes (Fig 1C and 1D). Additionally, when stratifying by TE changes (S5 Fig), the mRNAs with significant relative TE changes show more concordance in Ribo-Seq reads and protein abundance (S5B Fig, left) compared to either RNA-Seq reads and protein abundance (S5B Fig, right) or to the mRNAs with unchanged TE (S5A Fig, left). These results indicate that the absence of Pab1 affects the initiation process of most mRNAs to the same extent, such that the number of ribosomes recovered for a particular mRNA ORF remains proportional to the mRNA level. This observation is consistent with previous reports of human cells depleted of PABPC [40,41].

To further investigate how deletion of *PAB1* impacts translation initiation, we characterized 470 completely spliced mRNAs that showed significant increases (“Up”) or decreases (“Down”) in relative TE in response to *PAB1* deletion. We found that mRNAs with increased TE are those that are generally more abundant in the WT Pab1 condition (*pbp1Δ* cells) (Fig 5B, orange) but become relatively depleted upon *PAB1* deletion (Fig 5C, orange). Gene ontology analysis revealed that these mRNAs are related to metabolism, catabolism, biosynthetic process, and ribosomes (S2 Table and S6 Fig). On the other hand, mRNAs with decreased TE have average abundance in *pbp1Δ* cells (Fig 5B, purple) but become relatively more abundant in *pab1Δpbp1Δ* cells (Fig 5C, purple). These mRNAs are related to ion transmembrane transport and ion homeostasis (S2 Table and S6 Fig). These observations imply that there may be a mechanism to translationally upregulate or downregulate these mRNAs to compensate for the relative decrease or increase in mRNA levels, respectively, to prevent too much fluctuation of protein levels.

However, it is unclear whether the translation efficiency phenotypes characterized in *pab1Δpbp1Δ* cells follow from the loss of Pab1’s direct role in translation initiation, as we cannot rule out an indirect effect of *PAB1* deletion on translation initiation factor levels. As shown in the analysis of transcriptome and proteome changes in response to *PAB1* deletion, some initiation factors were reduced at both mRNA and protein levels (Fig 2A, top left). These disproportionate initiation factor levels could potentially cause a global reduction in translation and the appearance of unchanged relative TE. Notably, among the most reduced initiation factors are the two paralogs of eIF4G (eIF4G1/Tif4631 and eIF4G2/Tif4632), a binding partner of Pab1.

### Reduction of initiation factor levels confound the effects of *PAB1* deletion on translation efficiency

To determine whether the reduction in eIF4G level can be ruled out as a possible explanation for the observed translation efficiency phenotypes, we again focused on 470 completely spliced mRNAs that showed significant increases (“Up”) or decreases (“Down”) in relative TE in response to *PAB1* deletion and compared them with two published data sets. First, we investigated TE changes obtained from ribosome profiling and RNA-Seq data of cells depleted for eIF4G through a degron system inducible by growth media and temperature shifts [68]. We found that mRNAs in the Down group upon *PAB1* deletion also have overall lowered TE upon 2 hours of eIF4G depletion compared to the Up group and the general distribution in the transcriptome (Reference “Ref.” group) (Fig 5D, 2hr). This trend is even observed before eIF4G depletion and immediately after depletion was initiated (Fig 5D, preshift and 0hr), which is unsurprising because the degron strain showed decreased eIF4G levels even in uninduced condition [68]. These results suggested that relative TE changes observed upon *PAB1* deletion may be partially mediated by reduction in eIF4G level in *pab1Δpbp1Δ* cells. For the second analysis, we utilized RIP-seq data of mRNAs associated with immunoprecipitated (IP’d) TAP-tagged eIF4G and Pab1 [36]. If our data was indirectly influenced by the reduction of eIF4G level, we expected that mRNAs enriched in IP of eIF4G, implying their increased dependence on eIF4G for translation initiation, would be most sensitive to eIF4G reduction–they would have decreased TE (i.e., be found in our Down group) and there would be a negative correlation between fold-change in IP and fold-change in TE. Indeed, we found negative relationships between fold change in TE and fold-change in IP of both eIF4Gs, but not in IP of Pab1 (S7A Fig). We also found that mRNAs in the Down group had: i) higher enrichment fold-change for eIF4G1 and eIF4G2 IPs than those in the Up group or Reference group (Fig 5E), ii) an over-representation of mRNAs that were significantly enriched in IPs of eIF4G1 or eIF4G2 compared to the Reference group (Fig 5F, blue, and S3 Table), and iii) an under-representation of mRNAs that were significantly depleted in IPs of eIF4G1 or eIF4G2 compared to Reference group (Fig 5F, red, and S3 Table). On the other hand, mRNAs in the Up group had: i) lower enrichment fold-change for eIF4G1 or eIF4G2 than those in the Down or Reference group (Fig 5E) and had an over-representation of mRNAs that were significantly depleted in IPs of eIF4G1 or eIF4G2, implying their reduced inclination for cap-dependent initiation (Fig 5F, blue, and S3 Table). Notably, the fold-change enrichment in Pab1 IP does not differ among different TE groups (Fig 5E), although mRNAs in the Up group had an over-representation of mRNAs significantly enriched for Pab1 IP (Fig 5F and S3 Table). Overall, these results indicate that reduction in eIF4G levels cannot be ruled out as a possible explanation for the observed changes in TE when *PAB1* is deleted.

Despite the apparent effect of eIF4G on different mRNA subgroups, eIF4G still does not explain the entirety of the data. For example, although the mRNAs enriched in eIF4G2 IP are proportionally over-represented in the Down group compared to Reference (36% vs. 21%), 64% of the mRNAs in the Down group are those not highly dependent on eIF4G (Fig 5F). Thus, we further characterized these mRNAs by exploring their 5’-UTR features.

**Fig 6:**
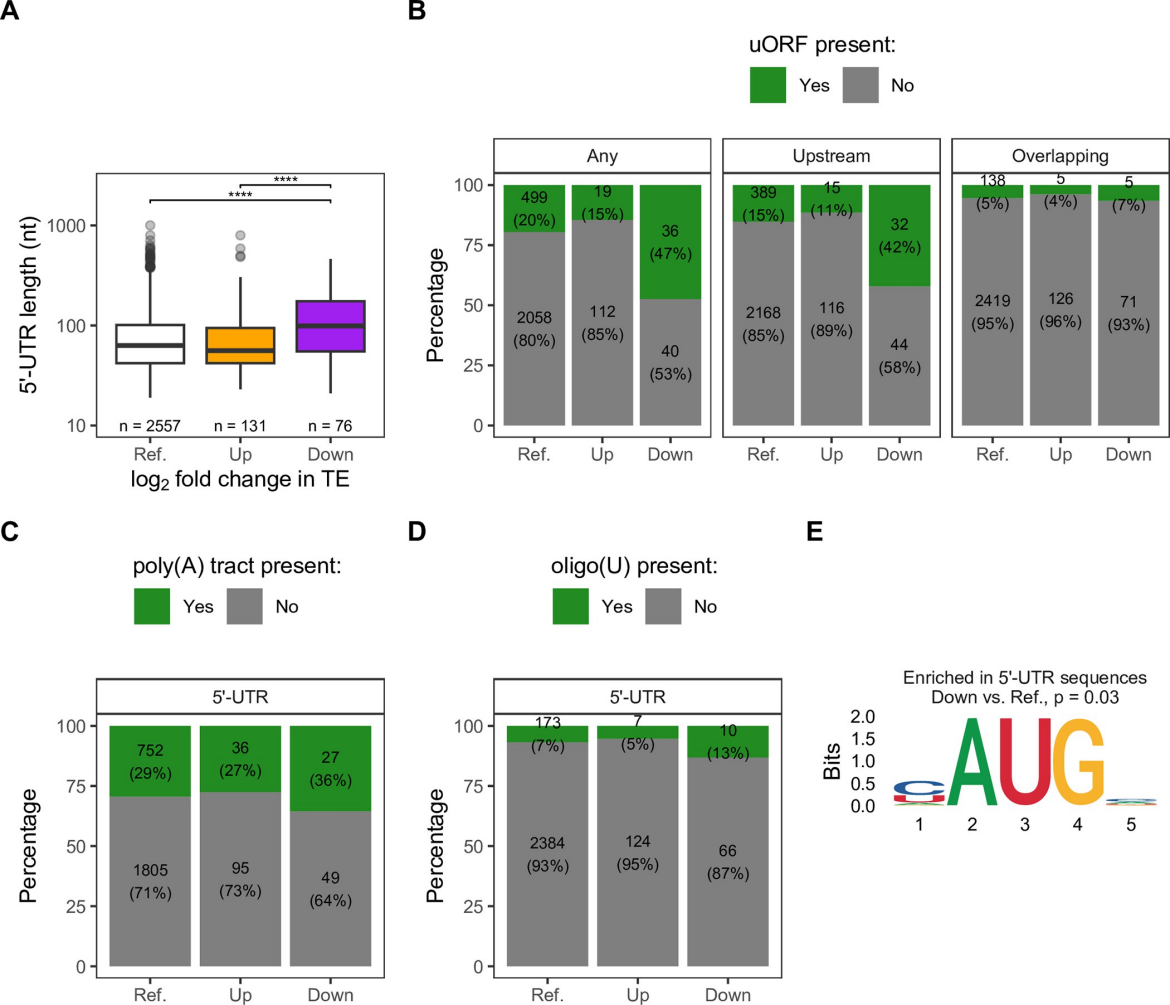
5’-UTR features of mRNAs with differential changes in translation efficiency. **A.** Distribution of 5’-UTR length of mRNAs in different TE groups from Fig 5A. Two-sided Wilcoxon’s rank sum test with Benjamini-Hochberg method for multiple-testing correction was used to compare values between pairwise groups. Only significant comparisons were reported as the following: (*) p < 0.05, (**) p < 0.01, (***) p < 0.001, (****) p < 0.0001. **B-D.** Proportion and number of mRNAs from groups in A with (“Yes”) or without (“No”) uORF (**B**), poly(A) tract (**C**), or oligo(U) (**D**) in the 5’-UTR. Pairwise Fisher’s exact test with Benjamini-Hochberg method for multiple-testing correction was used to compare between Reference, Up, and Down groups. p-values are provided in S3 Table. **E.** Motif enriched in 5’-UTR sequences of mRNAs in the Down group relative to Reference, identified by STREME. For all panels, analyses were limited to mRNAs with existing UTR annotations. Reference (Ref.) group includes all mRNAs regardless of TE changes (Up + Down + Unchanged) to recapitulate the general distribution of measured values in the transcriptome.

First, we observed that mRNAs in the Down group tend to have longer 5’-UTRs than those in the Up or Reference groups (Fig 6A). A longer 5’-UTR increases the chance for motifs or upstream open reading frames (uORFs) that could interfere with initiation complex assembly or the scanning mechanism. The presence of uORFs is generally thought to suppress translation initiation of the main ORF [69]. We found that the proportion of mRNAs with at least one uORF is higher in the Down group than in other groups (Fig 6B, left panel, and S3 Table). We further asked if the results were due to uORFs that are completely upstream or uORFs that are overlapping with the main ORF by conducting the same analysis for the two types of uORFs separately. The presence of overlapping uORFs is quite rare in our data set and the proportions are not different between groups, but the results for upstream uORFs mimicked those of all uORFs analyzed together (Fig 6B and S3 Table). Additionally, comparison of TE changes of mRNAs with uORFs to those without uORFs revealed that mRNAs with uORFs had a greater decrease in TE in all categories (S7B Fig). The sensitivity of uORF-containing mRNAs to *PAB1* deletion could be related to the relative reduction in eIF1 (Sui1) level in the *pab1Δpbp1Δ* strain (Fig 2A, top left). eIF1 has a role in start codon recognition, discriminating against suboptimal start sites in favor of the optimal one [70–73]. Therefore, it is not surprising that mRNAs with decreased TE tend to have uORFs (Fig 6B). Consistent with this notion, ribosome footprints in the 5’-UTR region are slightly increased in the *pab1Δpbp1Δ* strain relative to the other two strains (Fig 4B).

Next, we considered motif-dependent regulation of translation initiation. First, recruitment of Pab1 to poly(A) tracts in the 5’-UTR was reported to induce internal cap-independent initiation in yeast [74]. Therefore, mRNAs with this motif would be expected to have decreased TE in the absence of Pab1 and thus be found more often in our Down group. However, we did not find differences in the proportions of mRNAs containing poly(A) tracts in the 5’-UTR between groups (Fig 6C and S3 Table). Second, oligo(U) longer than 7 nt in the 5’-UTR was identified as eIF4G1’s preferential binding motif and can promote initiation [68,75]. However, we did not find differences in the proportions of mRNAs containing oligo(U) in the 5’-UTR between groups (Fig 6D and S3 Table). In a parallel approach, we used the motif discovery tool STREME [76] to identify motif(s) enriched in 5’-UTR sequences of either Up and Down group relative to the Reference. Neither poly(A) tract nor oligo(U) is enriched in either group compared to sequences from the Reference group, consistent with our direct analyses (Fig 6C and 6D), but the AUG motif is enriched in the Down group (Fig 6E), consistent with our uORF analysis (Fig 6B). No other novel motif was identified.

We also asked whether nucleotide context around the start codon and near the 5’ cap influence changes in TE in response to *PAB1* deletion by comparing proportions of nucleotides at each position from each group relative to Reference (S7C Fig). The optimal context for translation in yeast has been determined as AA(A/G)AAUGUCU, with position -3 (3^rd^ nucleotide upstream of AUG, which is considered as positions +1 +2 +3) being the most important and conserved [69,77,78]. We did not find mRNAs in the Up or Down group to have biases in nucleotide usage at position -3 compared to Reference or each other (S7C Fig, top). However, other positions in this window that show significant differences relative to the Reference are consistent with the consensus, namely, A/C are enriched in the Up group at position -4 (S7C Fig, top) and U is depleted in the Down group at position +4 (S7C Fig, middle). Beyond the immediate AUG context, G is enriched and U is depleted in the Down group at position +18 (S7C Fig, middle). From the 5’ cap, U is enriched and A is depleted in the Down group at position +6 (S7C Fig, bottom).

Overall, our results show that studying Pab1’s role on global translation initiation through deleting or depleting Pab1 can be confounded by the reduction in initiation factor levels, especially eIF4G and eIF1.

### Properties of the mRNAs with differential TE in response to *PAB1* deletion support the notion that mRNA 5’ and 3’ ends communicate

Changes in an mRNA’s poly(A) tail length can change the extent of its commitment to translation initiation, observations which led to the closed-loop model postulating that mRNA 5’ and 3’ ends communicate in translation [18,19,23,24]. Pab1 is thought to play an important role in facilitating the closed-loop mRNA structure, bridging the interaction with both the poly(A) tail and eIF4G [27–29]. Shorter mRNAs are thought to form more stable structures than longer mRNAs [28,79], but the closed-loop structure may not apply to every mRNA as not all mRNPs contain the closed-loop components [36,38,39] and for those enriched for closed-loop components, they have variable translation efficiency, not just high efficiency [39].

**Fig 7:**
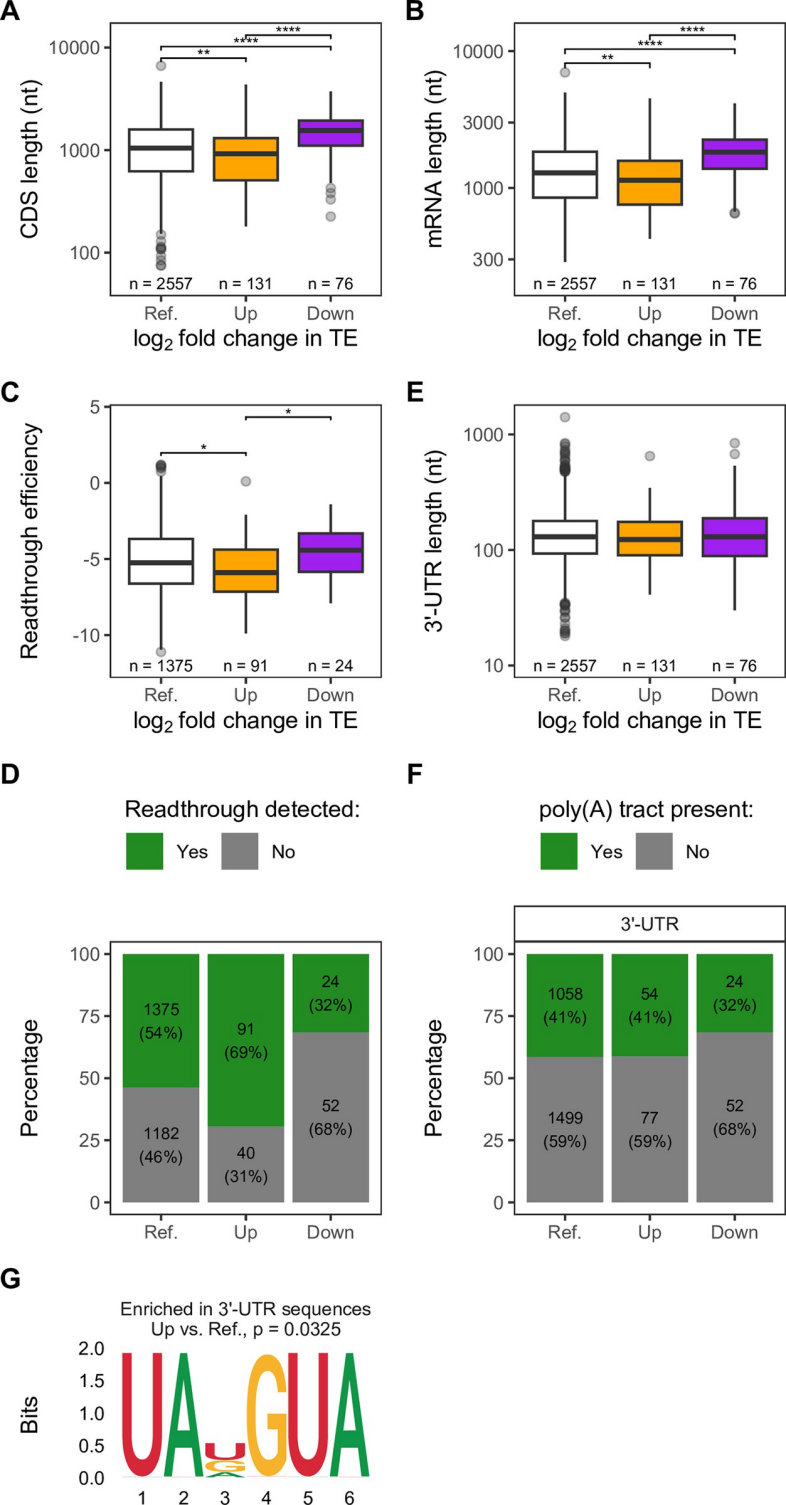
Evidence for communication of mRNA 5’-3’ ends in efficient initiation. **A-B.** Distribution of CDS length (**A**) or entire transcript length (**B**) of mRNAs in different TE groups from Fig 5A. **C.** Distribution of readthrough efficiency for mRNAs with detectable readthrough in the *pab1Δpbp1Δ* strain in each group. **D.** Proportion and number of mRNAs with detectable (“Yes”) or not detectable (“No”) readthrough in the *pab1Δpbp1Δ* strain. **E.** Distribution of 3’-UTR length in each mRNA group. **F.** Proportion and number of mRNAs with (“Yes”) or without (“No”) poly(A) tracts in the 3’-UTR. For A, B, C, and E, two-sided Wilcoxon’s rank sum test with Benjamini-Hochberg method for multiple-testing correction was used to compare values between pairwise groups. Only significant comparisons were reported as the following: (*) p < 0.05, (**) p < 0.01, (***) p < 0.001, (****) p < 0.0001. For D and F, Pairwise Fisher’s exact test with Benjamini-Hochberg method for multiple-testing correction was used to compare between groups. p-values are provided in S3 Table. **G.** Motif enriched in the 3’-UTR sequences of mRNAs in the Up group relative to Reference, identified by STREME. For all panels, analyses were limited to mRNAs with existing UTR annotations. Reference (Ref.) group includes all mRNAs regardless of TE changes (Up + Down + Unchanged) to recapitulate the general distribution of measured values in the transcriptome.

Efficient 5’-3’ communication allows efficient feedback of ribosomes recycled from termination to a new round of initiation, and this efficiency should be gene length-dependent, as diffusion of ribosomes between the ends would be expected to be proximity-based, i.e., more efficient for shorter mRNAs than longer mRNAs [80,81], even without Pab1 facilitating the closed-loop. This is especially relevant when the availability of ribosomes is limiting and ribosome recruitment becomes more dependent on recycled ribosomes than on limited free ribosomes [80,81], which may be the case in our data in light of the reduction in ribosomal protein levels in *pab1Δpbp1Δ* cells (Fig 2A). When comparing CDS and entire transcript lengths between groups, we found that mRNAs with decreased TE (Down group) tend to be longer than those with increased TE (Up group) and the Reference while those in the Up group tend be shorter (Fig 7A and 7B). These results are consistent with either the proximity-based or closed-loop 5’-3’ communication models.

To determine whether there is evidence for the closed-loop model, we stratified our transcript length analysis by whether the mRNAs were enriched or depleted in eIF4G or Pab1 RIP-seq data and whether their TEs were increased or decreased upon *PAB1* deletion (S8 Fig). At baseline (Ref. group), mRNAs depleted in eIF4G or Pab1 tend to be longer while those enriched tend to be shorter, with this trend strongest for Pab1 (S8 Fig), supporting the notion that closed-loop components are more likely to be associated with shorter transcripts [28,79]. However, in the absence of Pab1, mRNAs with decreased TE (Down group) tend to be longer mRNAs regardless of whether they were enriched for the closed-loop components or not (S8 Fig). This result suggests that the TE changes are likely independent of the closed-loop structure for these mRNAs. Additionally, this result is in line with our conclusion that, while mRNAs with decreased TE tend to be eIF4G-enriched (Fig 5E and 5F), eIF4G enrichment only makes up a subset of mRNAs with decreased TE and TE changes can be influenced by other mRNA features (Figs 6 and 7) that follow the proximity-based 5’-3’ communication model.

Consistent with the notion that efficient termination and recycling of ribosomes at the 3’ end promotes efficient translation initiation at the 5’ end, we found that mRNAs with increased TE have lower readthrough efficiency (i.e., more efficient termination—see next section and Methods for readthrough efficiency calculation) while those with decreased TE have higher readthrough efficiency (Fig 7C). We limited our analysis to mRNAs with detectable readthrough due to the cyclic nature of translation and the detection limit of readthrough ribosomes. Since the amount of readthrough ribosomes depends on the amount of translation of the CDS, for mRNAs with the same readthrough efficiency, readthrough may not be detectable for mRNAs with lower TE (so readthrough efficiency appears to be zero for them) but remain or become detectable for mRNAs with higher TE. Thus, the fact that the proportion of mRNAs with detectable readthrough in the Down group is lower than the Up group (Fig 7D and S3 Table) does not necessarily mean that readthrough efficiency is lower in the Down group. In sum, we found that mRNAs with increased TE had lower readthrough efficiency and those with decreased TE had higher readthrough efficiency (Fig 7C).

To see if Pab1’s function at termination influences the distinction between Up and Down TE groups in response to *PAB1* deletion, we compared 3’-UTR lengths among groups, but found no significant differences (Fig 7E). We next asked whether a specific sequence motif is enriched in either Up or Down group. No differences between groups were detected in terms of the presence of poly(A) tracts in the 3’-UTR region (Fig 7F and S3 Table), which can serve as a binding site of Pab1 in addition to the poly(A) tail, and this result was confirmed by the lack of poly(A) motif in the motif discovery approach, STREME. However, STREME identified the UAKGUA motif enriched in the Up group relative to Reference (Fig 7G). Although the probabilities for sense (UAU or UAC) and nonsense codons (UAA or UAG) were comparable for this motif, it is not impossible that the UAKGUA sequence may indicate two consecutive strong stop codons, where the second stop codon (although out-of-frame with the first) acts as a fail-safe stop codon in case of failed termination or recycling at the first stop codon. Moreover, the enrichment of G following the first stop codon is consistent with the observation that mRNAs with G at this position had the lowest readthrough efficiency (S10B Fig, “+4”). This motif being enriched in mRNAs in the Up group, along with the shorter mRNA length and lower readthrough efficiency of this group, is consistent with the hypothesis that more efficient ribosome recycling promotes efficient new rounds of translation initiation.

### Deletion of *PAB1* promotes transcriptome-wide accumulation of ribosomes downstream of normal stop codons

Deletion of *PAB1* has been shown to decrease translation termination efficiency and increase stop codon readthrough efficiency of reporter PTCs *in vivo* [51]. PTC readthrough occurs when a near-cognate tRNA outcompetes eRF1 in stop codon decoding, resulting in continued in-frame translation elongation and production of a C-terminally extended polypeptide [82,83]. Thus, we analyzed ribosome profiling data to determine whether decreased termination efficiency and increased readthrough efficiency could also be observed at normal termination codons (NTCs) of endogenous mRNAs when Pab1 is absent. Notably, the relative amount of ribosome footprints found in mRNA 3’-UTR regions is increased in *pab1Δpbp1Δ* cells compared to *pbp1Δ* or WT cells (Fig 4A inset and 4B), demonstrating that cells lacking Pab1 manifest an apparent termination defect. This defect occurred not only at canonical stop codons, as evidenced by a subtle increase in footprints in the “extension” region (the 3’-UTR region from the canonical stop codon to the next in-frame stop codon), but also at the first in-frame stop codons downstream of the canonical stop codon, as manifested by a significant increase in footprints in the distal 3’-UTR region (Fig 4B).

The presence of ribosomes in the 3’-UTR can arise from stop codon readthrough, ribosome frameshifting, or reinitiation. To determine the primary driver of increased ribosome footprints in the 3’-UTR of mRNAs in *pab1Δpbp1Δ* cells, analyses of reading frame proportions in different mRNA regions were carried out. Stop codon readthrough would yield footprints predominantly in reading frame 0 in the extension region, while frameshifting or reinitiation events would not show this bias. Indeed, the proportion of ribosome footprints in reading frame 0 increases in the extension region by 6.8% on average in *pab1Δpbp1Δ* cells compared to *pbp1Δ* or WT cells (Fig 4C) and this increase is comparable to that observed in ribosome profiling data of cells depleted of functional eRF1 (a 7% increase on average) [84,85]. Additionally, we found no correlation between 3’-UTR footprint density and fraction of out-of-frame footprints in the last 30 nt (10 codon) of the CDS (S9A Fig) as well as no significant difference in 3’-UTR footprint density between mRNAs with and without out-of-frame stop codons in the CDS (S9B Fig) in any strain, ruling out the possibility that out-of-frame translation that might have occurred in the CDS gave rise to increased footprints in the 3’UTR. Together, these results demonstrate that a notable portion of 3’-UTR footprints in *pab1Δpbp1Δ* cells arises from stop codon readthrough, thus suggesting that translation termination is less efficient in the absence of Pab1.

**Fig 8:**
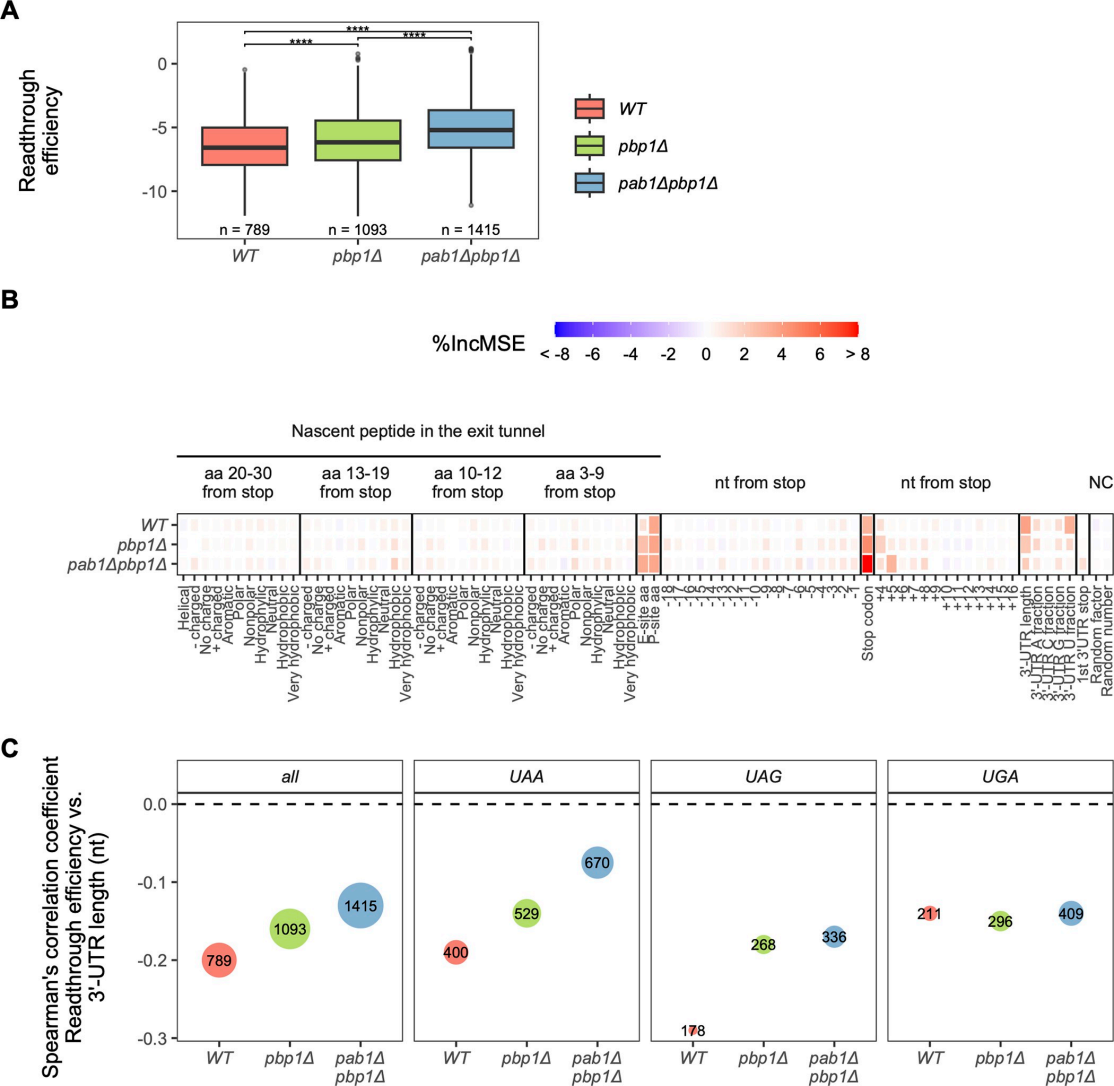
Pab1 regulates readthrough efficiency in a distance-dependent manner. **A.** Readthrough efficiency distribution in each strain (see Methods for calculation). Footprints from replicate libraries were pooled. Two-sided Wilcoxon’s rank sum test with Benjamini-Hochberg method for multiple-testing correction was used to compare values between pairwise strains. Only significant comparisons were reported as the following: (*) p < 0.05, (**) p < 0.01, (***) p < 0.001, (****) p < 0.0001. **B.** Average feature importance scores (percent increase in mean squared error (%IncMSE)) extracted from 25 random forest models (5-fold cross-validation, repeated 5 times) trained for each strain to predict readthrough efficiency. Higher feature importance score (red) means the prediction error is high when that feature is permuted. As negative controls (NC), each mRNA was assigned arbitrary continuous and discrete values (Random number and Random factor). Features with significant importance (empirical p-value < 0.05 in at least 15 out of 25 models) are represented as bigger tiles. **C.** Spearman’s correlation coefficient of the relationship between readthrough efficiency and 3’-UTR length using all available data (“all”) or split data by stop codon usage. Number and dot size reflect the number of mRNAs in each correlation coefficient. For all panels, only reads belonging to genes with UTR annotations and minimally overlapping sequences (less than 18 bp overlap with another gene on the same strand) were included in the analyses (2,693 genes).

To verify that the increase in stop codon readthrough is transcriptome-wide, i.e., that the results of Fig 4 were not derived from a limited number of mRNAs, we calculated readthrough efficiency for each mRNA by dividing the density of frame 0 footprints in the extension by that in the CDS region. The number of mRNAs with detectable readthrough is increased in *pab1Δpbp1Δ* cells, almost double that observed in WT, and overall readthrough efficiency in *pab1Δpbp1Δ* cells is significantly higher than in the other two strains (Fig 8A).

Curiously, the *pbp1Δ* strain also shows higher overall readthrough efficiency than the WT strain (Fig 8A), raising the question of whether Pbp1 plays a role in termination and contributes to increased readthrough in the *pab1Δpbp1Δ* strain. However, this observation is most likely not due to stop codon readthrough. Footprints in the 3’-UTR region in the *pbp1Δ* strain are only higher than in WT in the few codons immediately following the canonical stop codon, while in the *pab1Δpbp1Δ* strain, the increase in 3’-UTR footprints extend substantially beyond that (Fig 4A, inset). More importantly, the proportion of frame 0 footprints in the extension region in *pbp1Δ* cells does not differ from that in WT cells (Fig 4C). Although we limited our readthrough efficiency calculation to only frame 0 footprints to ensure as accurate a calculation as possible, we still cannot completely exclude other events, such as reinitation, that happened to also produce ribosome footprints in frame 0. Thus, despite the fact that deletion of *PBP1* did not result in proportionally higher stop codon readthrough events compared to WT, a slight increase in footprints immediately following the stop codon leads to an apparent increase in the calculated readthrough efficiency values in the *pbp1Δ* strain.

In short, *PAB1* deletion resulted in a translation termination defect, manifested as increased footprints in mRNA 3’-UTR regions. The preference for frame 0 footprints in the extension region and the higher median readthrough efficiency values calculated for individual mRNAs observed in *pab1Δpbp1Δ* cells compared to the two controls indicate that stop codon readthrough increases transcriptome-wide in the absence of Pab1.

### Release factor depletion is not the most likely explanation for increased ribosome footprints in mRNA 3’-UTRs upon *PAB1* deletion

Because PABPC plays major roles in the regulation of mRNA decay and translation, the termination defect and increased readthrough observed in *pab1Δpbp1Δ* cells can be due to: i) loss of Pab1’s direct function in termination via its interaction with eRF3 [45,52] or ii) changes in the stability of release factor mRNAs or changes in their translation that result in depletion of the respective proteins, which in turn affect global termination efficiency. Differential expression analyses on transcriptomic and proteomic data revealed that for the two release factors, eRF1 (Sup45) showed a slight reduction in its mRNA level but not its protein level, while eRF3 (Sup35) showed a slight but statistically significant reduction in both mRNA and protein levels in the absence of Pab1 (Fig 2A, bottom right), where the eRF3 protein level was 80% of that in *pbp1Δ* cells. However, the reduction in release factor levels may not necessarily result in reduced termination efficiency. If overall translation is also reduced, the normal stoichiometry of supply and demand for release factors may still be maintained or supply may even exceed demand. The latter conclusion follows from observations that PABPC depletion substantially reduces overall protein synthesis such that almost all heavy polysomes are lost [41,55] and protein synthesis is limited by the amount of free ribosomes [30,80,81,86]. Hence, since termination can only occur after initiation and elongation, the larger reductions in ribosomal proteins and initiation factors (the most reduced ribosomal protein and initiation factor are respectively reduced to 45% and 64% of their normal levels) would most likely be limiting and release factors would thus be expected to still be in excess.

We also considered whether increased ribosome footprints in the 3’-UTR arise from a reduced level of Rli1, a ribosome recycling factor. However, since recycling can only occur after termination, Rli1 may still be in excess stoichiometrically in the context of overall reduced translation in *pab1Δpbp1Δ* cells. More importantly, *pab1Δpbp1Δ* cells show a preference for frame 0 in the extension region (Fig 4C), consistent with stop codon readthrough, while Rli1 depletion cells resulted in reinitiation in the 3’-UTR in all three reading frames [87].

Termination occurs when the eRF1/eRF3 complex outcompetes the near-cognate aminoacyl-tRNA (aa-tRNA)/eEF1A complex in binding to the ribosomal A-site. Thus, in addition to the shift in equilibrium between the demand and supply of release factors, the shift in equilibrium between the levels of the eRF1/eRF3 complex and the aa-tRNA/eEF1A complex also affects termination efficiency. In *pab1Δpbp1Δ* cells, the relative level of eEF1A mRNA is unchanged but eEF1A protein is at 129% relative to the level in *pbp1Δ* cells (Fig 2A, top right). This increase in eEF1A to eRF3 ratio may increase the chance of stop codon decoding by aa-tRNA/eEF1A, reducing termination efficiency and increasing ribosome footprints in the 3’-UTR regions.

Together, previous studies and our results here suggest that a significant reduction in translation is reducing the demand for release factors and recycling factors, making them likely to still be in excess. Therefore, the observed decreased termination efficiency or increased stop codon readthrough in *pab1Δpbp1Δ* cells may not be because the release factor levels are limiting but could be explained by an increase in competitive advantage of aa-tRNA/eEF1A in stop codon decoding, in addition to a loss of Pab1’s stimulatory function on termination.

### 3’-UTR length is no longer predictive of readthrough efficiency when *PAB1* is deleted

Pab1 has been shown to affect NMD-sensitivity and readthrough efficiency of PTC-containing mRNAs in a manner dependent on PTC proximity to mRNA-associated Pab1 [48,51,79]. As would be expected from this relationship, readthrough of PTCs in reporter mRNAs increased in response to 3’-UTR lengthening, but this trend was lost when *PAB1* was deleted [51]. Recently, we investigated the *cis*-regulatory elements of transcriptome-wide stop codon readthrough using a random forest machine learning approach and found that 3’-UTR length was an important predictor of readthrough, where mRNAs with short 3’-UTRs had lower readthrough than those with long 3’-UTRs when eRF1’s functionality was compromised, but the trend was the opposite in WT cells [84]. These data led us to further assess the involvement of Pab1 and its proximity to the stop codon as a predictor of readthrough efficiency. If Pab1 is involved, we expected that the relationship between readthrough efficiency and 3’-UTR length would disappear or weaken when *PAB1* is deleted. Thus, we applied the same random forest approach to identify mRNA features that influence the prediction of readthrough efficiencies in WT, *pbp1Δ*, *pab1Δpbp1Δ* strains (Figs 8B and S10A). As expected, the negative control features (NC) have no influence on readthrough efficiency prediction in any strain, while the identity of the stop codon is an important predictor of readthrough in all strains (Fig 8B). The length of the 3’-UTR is an important predictor of readthrough efficiency in WT and *pbp1Δ* cells but is no longer important in *pab1Δpbp1Δ* cells (Fig 8B). The relationship between 3’-UTR length and readthrough efficiency is slightly negatively correlated in WT and *pbp1Δ* strains, but it is weakened in the *pab1Δpbp1Δ* strain (Fig 8C, “all”). The fact that the correlation is not completely eliminated even in the absence of Pab1 could be due to technical limitations of readthrough efficiency calculation, where frame 0 non-readthrough 3’-UTR footprints were inevitably included. Nevertheless, when other mRNA features were included in the analysis and controlled for (i.e., random forest regression model), this weak correlation became insignificant in predicting readthrough efficiency in *pab1Δpbp1Δ* cells.

To further see how 3’-UTR length synergistically regulates readthrough with the strongest feature, stop codon identity, we grouped mRNAs by their stop codon identities and then performed the correlation analysis (Fig 8C). We found that the correlation between readthrough efficiency and 3’-UTR length is closer to zero in the absence of Pab1 than those observed in the other two strains for mRNAs with UAA as the stop codon, which happens to be the most common stop codon in the yeast transcriptome, but this trend isn’t observed for UAG and UGA (Fig 8C). This result suggests that mRNAs with UAG and UGA, although allowing higher readthrough (Fig S10B), are less sensitive to the proximity of Pab1 to the stop codon in this readthrough measurement, possibly because termination is slower and rate-limiting, unlike UAA where termination is faster. Since stop codon identity is a more important feature affecting readthrough efficiency, the effect caused by loss of Pab1 is somewhat masked.

Overall, we find that the proximity of Pab1 to the stop codon, as measured by 3’-UTR length, plays a role in readthrough efficiency prediction in combination with other known mRNA features in cells with WT Pab1 conditions (WT and *pbp1Δ* cells). The biological explanation for the inverse relationship between 3’-UTR length and readthrough efficiency is still unknown in WT cells. However, because 3’-UTR length was still predictive of readthrough efficiency in cells depleted of functional eRF1 [84], which also increased transcriptome-wide readthrough, the fact that 3’-UTR length no longer predicts readthrough of mRNAs in *pab1Δpbp1Δ* cells supports the notion that readthrough occurring in *pab1Δpbp1Δ* cells is likely due to loss of Pab1’s direct function in termination rather than reduction in functional release factor levels.

## Discussion

Numerous biochemical, structural, *in vitro*, and *in vivo* studies of specific mRNAs have identified pleiotropic roles for PABPC in cytoplasmic mRNA deadenylation, translation initiation, and translation termination [3]. However, because of PABPC’s multiple apparent functions, defining its transcriptome-wide roles has been difficult. High-throughput approaches exploring transcriptome-wide effects of PABPC depletion have been carried out in mammalian cells [40,41], but none have been done in yeast. Consistent with observations in mammalian cells, we found that deletion of yeast *PAB1* resulted in major changes in the transcriptome (Fig 1B), but only minimal changes in relative translation efficiency (TE) (Fig 5A). We showed that *pab1Δ* cells also drastically changed their proteome (Fig 1A). The significant reduction in ribosomal proteins suggests that global translation rates would decrease, yet we find TE of most mRNAs to be unchanged (Fig 5A), indicating that global mRNA levels are also decreased as a consequence of *PAB1* deletion. Further, we provided the first evidence for a transcriptome-wide translation termination defect (Figs 4 and 8A). These results demonstrated the vast pleiotropic aspects of *PAB1* deletion.

Our proteomics data have provided insights to the direct vs. indirect consequences of *PAB1* deletion and suggested a new layer of complexity to interpreting genome-wide gene expression alterations in PABPC-depleted cells. PABPC’s role in translation initiation has been elusive, partly because the extent of its activity is dependent on the stoichiometry between PABPC, poly(A) tracts, and basal translation levels [40], and these experimental conditions frequently vary between studies. Moreover, Pab1 appears to have preferential association with certain mRNAs [36]. Hence, it seems counterintuitive that depleting PABPC/deleting *PAB1* reduces translation overall, yet relative TE is unchanged for most mRNAs [40,41]. These observations are akin to the effects of depleting eIF4G in yeast [88]. Since *PAB1* deletion also reduced eIF4G mRNA and protein levels (Fig 2A), it is possible that effects attributed to the absence of Pab1 are at least partially due to dysregulation of eIF4G mRNA stability and the consequent reduction of eIF4G, which in turn reduced global translation initiation. This sequence of events is also likely for ribosomal proteins, as PABPC depletion has been shown to cause accelerated decay of mRNAs with short poly(A) tails [40], which usually are characteristics of highly expressed, highly translated mRNAs, including those encoding ribosomal proteins [3,89]. Even when we focused our analyses on mRNAs that did have significant changes in TE, where their drastic changes may be due to specific factors outside of the global regulators of translation initiation, their properties are still related to eIF4G-dependent pre-initiation complex recruitment, eIF1-mediated start codon recognition, and efficiency of ribosome recycling (Figs 5–7). Due to their intrinsic properties, these mRNAs with significant increase or decrease in TE are respectively more or less dependent on these processes than most mRNAs and are thus more sensitive to reduction in these factors in *pab1Δ* cells (Fig 2A). As a result, the direct role of Pab1 in initiation is masked by the changes in these core components of initiation, i.e., reduction of initiation factor levels cannot be completely ruled out as an explanation for the translation initiation defects observed in *pab1Δ* cells. It is also possible that the role of Pab1 in initiation is masked by the strain background used in our study, where yeast cells may have already altered their gene expression profiles to compensate for the lack of Pab1 over extended period of time upon full gene deletion and reached a steady state, because more widespread TE changes (~2.5 times more transcripts than our study) have been observed in a yeast strain in which Pab1 was degraded over a short period of time (6 hours) by the inducible degron system [90]. Nevertheless, this recent study also found that TE changes observed under a limiting Pab1 level seem to be an indirect consequence of another phenomenon, albeit from a different aspect from our study, namely the accelerated mRNA degradation process [90].

Indirect consequences of *PAB1* deletion through reduced level of key pathway components might be applied to translation termination as well, since there is a slight reduction in release factor levels in *pab1Δ* cells (Fig 2A). The increased stop codon readthrough observed in *pab1Δ* cells is unlikely to be due to the release factor level becoming limited, since reduced initiation factors and ribosomal proteins are more limiting than release factors, skewing the usual stoichiometry of demand vs. supply for release factors towards the supply, but this observation is likely due to a shift in stoichiometry of eRF1/eRF3 vs. aa-tRNA/eEF1A towards aa-tRNA/eEF1A, promoting readthrough. However, Pab1’s direct role in termination is still implied because i) 3’-UTR length, which approximates the distance of Pab1 to the stop codon, lost its ability to predict readthrough efficiency in *pab1Δ* cells as opposed to WT (Fig 8B) or eRF1 mutant cells [84] (i.e., 3’-UTR length should have still been predictive of readthrough efficiency if increased readthrough was a consequence of release factor depletion alone), and ii) deletion of Pab1’s eRF3 interacting domain only (*pab1ΔC*), which affects termination but not mRNA decay [45], yields significant stop codon readthrough of reporter mRNAs [51]. Nevertheless, it remains to be determined whether a 20% reduction in termination factors in a *PAB1* strain would yield the same termination defect as a *pab1Δ* mutation and whether *pab1ΔC* cells significantly change their release factor levels. Because near cognate tRNAs compete with release factors in stop codon decoding, changes in aminoacyl-tRNA levels, synthesis, and modifications should also be considered.

Since deletion of the *PAB1* gene in yeast is lethal, we also deleted *PBP1*, which suppresses this lethality. Surprisingly, single deletion *pbp1Δ* cells showed an apparent increase in readthrough efficiency compared to WT cells (Fig 8A), raising a question whether Pbp1 plays a role in translation termination. However, this is unlikely for several reasons. First, we noticed that the amount of footprints in the 3’-UTR from *pbp1Δ* cells is only relatively higher than WT in the first few codons after the stop codon, and from only one of the three biological replicates, compared to *pab1Δpbp1Δ* cells in which the higher amount of footprints sustains throughout the 80-nt window (Fig 4A). This observation is clearer through quantifications (Fig 4B). Second, footprints in the extension region from *pbp1Δ* cells do not show frame 0 preference as do those from *pab1Δpbp1Δ* cells (Fig 4C). Despite the lack of frame 0 preference, these footprints unfortunately cannot be selectively discarded for readthrough efficiency calculation, defined as frame 0 footprint density in the extension relative to that in the CDS. Thus, by this definition, the calculation results in an apparent increase in readthrough efficiency in *pbp1Δ* cells (Fig 8A). It is important to note that the same apparent “increase” in “readthrough efficiency” was also observed in cells lacking recycling factor Rli1 [84], which caused random reinitiation downstream of stop codons and not readthrough [87].

We showed that the sets of mRNA substrates of Pat1/Lsm1-mediated decay and NMD both manifest relative increases in abundance and thus likely to be partially stabilized in *pab1Δ* cells despite their different decay mechanisms, while substrates of Dhh1-mediated decay did not have a tendency to increase or decrease (Fig 3). Stabilization of Pat1/Lsm1 substrates may be attributable to the loss of Pab1’s role in stimulating deadenylation of mRNAs usually subject to deadenylation-dependent decay. However, there is also a slight depletion of Ccr4-Not components, Not4 and Not5 (Fig 2B), and the extent of how much this depletion contributes to substrate stabilization is unknown. The consequences of Not4 and Not5 depletion may also apply to some Dhh1 substrates (i.e., those that are increased) but not all, possibly due to Dhh1’s involvement in multiple decapping complexes which can lead to alternative degradation pathways [9]. Some complexes may require Pab1’s role in deadenylase recruitment while for others, Dhh1’s communication with the Ccr4-Not complex may be enhanced by lower Pab1 level [65]. In contrast to Pat1/Lsm1 substrates, partial increases in the levels of NMD substrates most likely arises from the combination of decreased translation and decreased Upf1. The reduction in overall translation would result in fewer instances of termination and, together with the slight reduction in Upf1, would lower the likelihood that a nonsense-containing mRNA would be targeted by NMD.

Collectively, our observations of the pleiotropic effects of *PAB1* deletion may help explain the discrepancies in previous studies of PABPC’s functions and our multi-omics data can be helpful resources for the design of future experiments involving genetic manipulation, depletion, or overexpression of PABPC.

## Materials and methods

### Yeast strain construction

Yeast strains used in this study, listed in S4 Table, are in the W303 background. Gene deletions were achieved by the PCR-based method [91] and high efficiency transformation [92] of fragments amplified by oligonucleotides listed in S5 Table, synthesized by Integrated DNA Technologies (IDT). Three biological replicates (isolates) of each mutant strain and three technical replicates of isogenic wild-type (WT) strain were used for all experiments.

The *pbp1Δ* strain was made by replacing the coding sequence of the *PBP1* gene in a WT strain (HFY114) with the *URA3* gene. The *URA3* cassette was obtained from plasmid HFSE1380 [59] by PCR using URA3_5F_v2 and URA3_3R_v2 primers. Homology arms flanking the *PBP1* coding sequence were amplified from genomic DNA by PCR using PBP1_5F, PBP1_5R_v2, PBP1_3F_v2, and PBP1_3R primers. DNA fragments consisting of the homology arms flanking the *URA3* cassette were constructed by PCR and transformed into competent WT yeast cells. Verification of successful gene replacement was confirmed by PCR followed by Sanger sequencing using primers listed in S5 Table.

Subsequent deletion of the *PAB1* gene from the *pbp1Δ* strain was carried out by replacing the coding sequence of *PAB1* with kanMX, resulting in a *pab1Δpbp1Δ* strain. The kanMX cassette was obtained from the pCAS plasmid (Addgene, #60847) by PCR using PAB1_KanMX_F and PAB1_KanMX_R primers. Homology arms flanking the *PAB1* coding sequence were amplified from genomic DNA by PCR using PAB1_5H_ext_F2, PAB1_5H_ext_R, PAB1_3H_ext_F, and PAB1_3H_ext_R primers. DNA fragments consisting of the homology arms flanking the kanMX cassette were constructed by PCR and transformed into competent *pbp1Δ* (KMY01) yeast cells. Verification of successful gene replacement was confirmed by PCR followed by DNA sequencing using primers listed in S5 Table as well as by examining growth phenotype. Doubling time for these strains grown in YEPD at 30°C were 1.5 hours for WT and *pbp1Δ* strains and 3.5–4.5 hours for the *pab1Δpbp1Δ* strain.

### Cell growth and harvest

Cells were grown in 1 L of YEPD at 30°C with shaking. When the OD_600_ of the culture reached 0.6–0.8, cells were collected by rapid vacuum filtration, flash-frozen in liquid nitrogen in the presence of Footprinting Buffer (20 mM Tris-HCl pH 7.4, 150 mM NaCl, 5 mM MgCl_2_) plus 1% TritonX-100, 0.5 mM DTT, 1 mM phenylmethylsulfonyl fluoride (PMSF), and 1X protease inhibitors, and lysed in a Cryomill (Retsch) (5 Hz, 2 min; 10 Hz, 15 min). Cell lysates were clarified by ultracentrifugation in a Beckman Coulter Optima L-90K Ultracentrifuge at 18,000 rpm for 10 min at 4°C, using a 50Ti rotor. Centrifugation was repeated for the supernatant at 18,000 rpm for 15 min at 4°C. Lysates were stored at -80°C in aliquots.

### Ribosome profiling library preparation and sequencing

Ribosome profiling libraries were prepared as described previously [93]. Lysates were digested with RNase I (Invitrogen, #AM2294) for 1 hour at 25°C with shaking at 700 rpm, and the reaction was stopped using SUPERase-In RNase Inhibitor (Invitrogen, #AM2694). RNase I-treated lysates were then layered onto a 1 M sucrose cushion in Footprinting Buffer plus 0.5 mM DTT and centrifuged in a Beckman Optima TLX Ultracentrifuge at 60,000 rpm for 1 hour at 4°C using a TLA100.3 rotor to isolate 80S ribosomes. Ribosome-protected fragments (RPFs) were extracted from pelleted 80S ribosomes using a miRNeasy kit (QIAgen, #217004) following the manufacturer’s protocol for enriched recovery of small RNAs (<200 nt). RNAs larger than 200 nt, which include ribosomal RNAs (rRNAs) and other large RNAs, were discarded. Small RNAs were 3’ dephosphorylated and 5’ phosphorylated with T4 polynucleotide kinase (T4PNK, NEB, #M0201S), and purified with RNA Clean and Concentrator-5 (Zymo Research, #R1013) according to the manufacturer’s instructions that separately recover small and large RNA fractions. Large RNA fractions were discarded. Approximately 1 μg of small RNAs in 8.5 μl were incubated with 2 μl of QIAseq FastSelect–rRNA Yeast Kit (Qiagen, #334215) at 75°C, 2 min; 70°C, 2 min; 65°C, 2 min; 60°C, 2 min; 55°C, 2 min; 37°C, 2 min; 25°C, 2 min; 4°C, hold. This step hybridized any remaining rRNAs in the sample to the rRNA oligonucleotides, creating duplexes that would fail to ligate to sequencing adapters or fail to be reversed-transcribed into cDNA for sequencing library preparation. Sequencing libraries were prepared from the 10.5 μl reactions using the NEXTflex Small RNA-Seq Kit v3 (Perkin Elmer/Bioo Scientific, #NOVA-512-05) according to the manufacturer’s protocol, except for the RNA denaturation step (70°C, 2 min incubation) before 3’ 4N Adenylated Adapter ligation, which was skipped. Based on the manufacturer’s instructions for optimization, adapters were undiluted, PCR was performed for 15 cycles, and the library was purified using the manufacturer’s gel-free size-selection and cleanup protocol. Extra rounds of cleanup were performed if the amount of PCR primers was still high compared to the amount of library, as analyzed on a Fragment Analyzer. Three libraries were multiplexed according to NEXTflex’s recommended combinations of barcodes (index sequences) and sequenced (single-end, 75 cycles) in-house on Illumina NextSeq 500 or NextSeq 550 sequencers.

### RNA-Seq library preparation and sequencing

RNA-Seq libraries were prepared and sequenced as described previously [93]. Briefly, total RNAs were extracted from lysates using a miRNeasy kit (QIAgen, #217004) following the manufacturer’s protocol for recovery of total RNAs (standard protocol). Genomic DNA contamination was depleted using Baseline-Zero DNase (Lucigen/Epicentre, #DB0715K) according to manufacturer’s instructions. Approximately 1 μg of DNase-treated RNAs were used to prepare a sequencing library. The rRNA depletion strategy using QIAseq FastSelect–rRNA Yeast Kit (Qiagen, #334215) was integrated into the RNA fragmentation step of the TruSeq Stranded mRNA Library Prep kit (Illumina, #20020594) according to the QIAseq FastSelect’s manual. Three libraries were multiplexed using recommended combinations of TruSeq RNA Single Indexes Set A (Illumina, #20020492) and sequenced (single-end, 75 cycles) on an Illumina NextSeq 500 sequencer.

### Sequence alignment

The yeast transcriptome used for sequence alignment was from https://github.com/Jacobson-Lab/yeast_transcriptome_v5, the generation of which was described previously [84]. Briefly, gene annotations were downloaded from the Saccharomyces Genome Database (https://www.yeastgenome.org; September 10, 2015). For UTR information, the longest UTR entry was chosen for mRNAs with multiple annotations across studies [94–98], which were downloaded from the YeastMine database (July 3, 2017). Reads pre-processing, alignment, and quantification were performed on the University of Massachusetts Green High Performance Computing Cluster using the following provided software packages: cutadapt v1.9, bowtie v1.0.0, fastqc v0.10.1, samtools v0.1.19, bedtools v2.26.0, UMI-tools v1.1.1, and RSEM v1.3.0.

Ribosome profiling reads were pre-processed, aligned to the transcriptome, and transcript abundance quantified as described previously, except for the PCR duplicate removal step, where the UMI-tools software package was employed [99]. The UMI-tools’ “extract” function was used to record four nucleotides at each end of a read, which were introduced during library preparation by the NEXTflex Small RNA-Seq Kit v3. UMI-tools’ “dedup” function with the default (“directional”) method was used to identify and remove PCR duplicates based on the extracted UMIs. Number of reads processed, number of PCR duplicates removed, number of remaining unique reads, and other relevant sequencing statistics for each library are provided in S6 Table.

RNA-Seq reads were aligned to the transcriptome and transcript abundance quantified using RSEM without any pre-processing.

### Mass spectrometry (LC-MS/MS)

#### Sample preparation

Protein concentrations in cell lysates were determined by Pierce BCA Protein Assay according to the manufacturer’s protocol (Thermo Scientific). Aliquots of cell lysates containing 50 μg total protein were snap frozen, lyophilized in a SpeedVac, then reduced, alkylated, and digested following the S-Trap digestion protocol (ProtiFi). In brief, lyophilized lysates were first resuspended in 23 μl Lysis buffer (5% SDS in 50mM Triethyl ammonium bicarbonate (TEAB)). Resuspended protein extracts were reduced by adding 1 μl 200mM TCEP and incubating at 55°C for 1 hour, then alkylated by adding 1 μl 375mM iodoacetamide (IAA) and incubating at room temperature for 30 minutes, protected from light. To further denature and trap proteins, samples were mixed with 2.5 μl of 27.5% phosphoric acid (H_2_PO_4_ in water) and 165 μl of binding/wash buffer (100mM TEAB in 90% methanol). The mixtures were applied to S-Trap columns and centrifuged at 4,000 g for 30 seconds. Columns were washed 5 times, each by 150 μl of binding/wash buffer and centrifugation at 4,000 g for 30 seconds. To digest proteins, 25 μl of digestion buffer containing 1 μg trypsin (5 μl of 0.2 μg/μl trypsin in 50mM TEAB + 20 μl 50mM TEAB) was added to each column and samples were incubated at 37°C overnight. Digested peptides were collected by 3 subsequent centrifugations at 4,000 rpm for 1 min following the addition of these elution buffers for each collection: 1) 40 μl 50mM TEAB in water, 2) 40 μl 0.2% formic acid in water, and 3) 40 μl 50% acetonitrile in water. All flowthroughs from the same sample were pooled, lyophilized in a SpeedVac, and stored at -80°C.

Samples were labeled using a TMT10plex labeling kit (Thermo Scientific). Lyophilized, digested peptides were resuspended in 50 μl 100mM TEAB and incubated with 10 μl of TMT10plex reagent (equilibrated to room temperature and resuspended in acetonitrile) at room temperature for 1 hour. Reactions were quenched with 5 μl of 5% hydroxylamine at room temperature for 15 minutes. Equal amounts (55 μl) of each sample were pooled together; 50 μl of the pooled reaction was saved for direct shotgun analysis and the rest for high-pH fractionation. For the latter, pooled samples were dried in a SpeedVac, resuspended in 300 μl of 0.1% trifluoroacetic acid (TFA) in water, and fractionated using a Pierce High pH Reversed-Phase Peptide Fractionation Kit (Thermo Scientific), collecting 1 flowthrough fraction, 1 wash fraction, and 6 step-gradient fractions.

#### Data acquisition

Mass spectrometry data was acquired using an Orbitrap Fusion Lumos Tribrid Mass Spectrometer (Thermo Scientific). Dried peptides were resuspended in 18 μl of 5% acetonitrile with 0.1% formic acid in water, vortexed for 2 minutes, and centrifuged at 16,000 rpm for 16 minutes. For mass spectrometry, 3.8 μl of the resuspended peptides were injected into the Mass Spectrometer. Peptides were trapped for 4 minutes at a flow rate of 4.0 μl/min onto a 100 μm I.D. fused-silica precolumn (Kasil frit) packed with 2 cm of 5 μm ReproSil-Pur 120 C18-AQ (dr-maisch.com), and eluted and separated in 120 minutes at a flow rate of 300 nl/min by an in-house made 75 μm I.D fused silica analytical column (gravity-pulled tip) packed with 25 cm of 3 μm ReproSil-Pur 120 C18-AQ (dr-maisch.com). Mobile phases were A (water (0.1% (v/v) formic acid) and B (acetonitrile (0.1% (v/v) formic acid). The biphasic elution program was as follows: 0–100 min (10–35% B); 100–120 min (35–65% B); 120–121 min (65–95% B); 121–126 min (95% B); 126–127 min (95–5% B); 145 min (STOP).

The MS data acquisition was performed in positive electrospray ionization mode (ESI+), within the mass range of 375–1500 Da with the Orbitrap resolution of 120,000 (*m/z* 200) and a maximum injection time of 50 milliseconds. Data dependent acquisition (ddMS2) was carried out with a 1.2 Da isolation window, a resolution of 30,000 (*m/z* 200), maximum injection time of 110 milliseconds, and the customed AGC target with a 38% of HCD collision energy.

### Data analysis

Raw data files were processed with Proteome Discoverer (version 2.1.1.21, Thermo Scientific) and searched against the Uniprot Saccharomyces cerevisiae database (downloaded 06/28/2021) using Mascot Server (version 2.8, Matrix Science). Search parameters included full trypsin, with variable modifications of oxidized methionine, pyroglutamic acid (from Q), and N terminal acetylation. Fixed modifications were carbamidomethylation on cysteine and TMT10plex on peptide N-terminus and lysine side chain. Assignments were made using a 10ppm mass tolerance for the precursor and 0.05 Da mass tolerance for the fragments. Peptide and protein validation and annotation was done in Scaffold (version 5, Proteome Software, Inc.) using Peptide Prophet [100] and Protein Prophet [101] algorithms. Peptides were filtered at a 1% FDR, while protein identification threshold was set to greater than 99% probability and with a minimum of two identified peptides per protein. Protein clustering analysis was applied to increase the probability of protein identification for proteins that share peptides (e.g. paralogs). Quantitative analyses, including TMT label-based quantification, median normalization of log_2_ intensity values, and log_2_ fold change calculation were carried out in Scaffold Q+S.

Data acquired from flowthrough and wash fractions were used to determine the success of high-pH fractionation. Further analysis was based on data acquired from 6 step-gradient fractions.

### Bioinformatics and statistical analyses

Data analyses and visualization were performed in the R software environment versions 3.5 and 4.2 using the following R packages: data.table, dplyr, reshape2, readxl, openxlsx, caret, randomForest, rfPermute, rstatix, rcompanion, DESeq2, limma, Biostrings, seqinr, riboWaltz, ORFik, gprofiler2, scales, ggplot2, ggpubr, ggh4x, ggrepel, ggVennDiagram, ggseqlogo, patchwork, and Cairo.

#### Ribosome profiling analysis and readthrough efficiency calculation

Aligned reads of ribosome profiling libraries were processed by R package riboWaltz [102] for initial diagnostic, read length filter (retain reads 20–23 nt and 27–32 nt in length), and determination of read’s P-site offsets, which were manually checked and modified for accuracy (S7 Table). mRNA regions (5’-UTR, CDS, extension, and distal 3’-UTR) and their lengths were adjusted accordingly to consider the stop codon as part of the 3’-UTR. Read counts belonging to different mRNA regions, read’s reading frame, and metagene analysis assessing periodicity were based on the read’s P-site location of mRNAs with annotated UTRs.

Readthrough efficiency was calculated for each mRNA as follows:

$$Readthrough\;efficiency=\frac{frame\;0\;read\;count\;in\;extension/length\;of\;extension\;(nt)}{frame\;0\;read\;count\;in\;CDS/length\;of\;CDS\;(nt)}$$

where the first 15 bp of the CDS region were excluded to avoid bias in ribosome accumulation over or near the start codon, and the extension region was defined as the 3’-UTR region from the canonical stop codon (inclusive) to the next in-frame stop codon (exclusive). mRNAs with RPKM of the CDS > 0.2 and RPKM of the extension > 0.1 were used for all readthrough efficiency analyses.

#### Random forest models

Random forest analyses were carried out with R packages caret [103], randomForest [104,105], and rfPermute [106]. For each sample, a random forest regression approach was trained to use mRNA features (previously defined in Mangkalaphiban et al. 2021 [84]) to predict readthrough efficiency values with 5-fold cross-validation, repeated 5 times, resulting in a total of 25 models. Each model was trained with 100 trees, the default number of features to split at each tree node (square root of number of features), and 1,000 permutation replicates to empirically determine p-value for feature importance. Feature importance score, percent increase in mean squared error (%IncMSE), was an average of scores extracted from 25 models. A feature was considered significantly important predictor of readthrough efficiency if its empirical p-value was less than 0.05 in at least 15 out of 25 models. Model performance metric was reported as an average of root mean squared error normalized to the range of readthrough efficiency values (NRMSE) across 25 regression models.

#### Analysis of transcript abundance changes

All analyses involving transcript abundance changes were performed with the R package DESeq2 [107]. The “expected_count” columns in the RSEM file output “isoforms.results” were used as input raw read count. Results were extracted with automatic independent filtering applied at significant cutoff (alpha) of 0.01. The false discovery rate (FDR) method was used to adjust the P-value.

For differential expression analysis of RNA-Seq or Ribo-Seq libraries, mRNAs with adjusted P-value < 0.01 were considered significantly differentially expressed between samples, regardless of magnitude of log_2_ fold change. For Figs 1C–1D, S2C–S2D, and S5, where RNA-Seq or Ribo-Seq log_2_ fold change were plotted against mass spectrometry log_2_ fold change, expected_count of mRNAs in the same protein cluster were added together and differential expression analysis was carried out as described.

For relative changes in translation efficiency (TE), ribosome profiling reads whose P-site locations were in the 5’-UTR, 3’-UTR, or the first 15 bp and the last 3 bp of the CDS (ribosomes paused over canonical start codon, translational ramp, and canonical stop codon) were discarded and transcript abundance for the rest of reads was re-quantified by RSEM. TE was defined as Ribo-Seq reads in CDS normalized to RNA-Seq reads. mRNAs with adjusted P-value < 0.05 were considered to have significant changes in TE between samples, regardless of the magnitude of log_2_ fold change.

#### Analysis of protein abundance changes

Differential abundance analyses of protein levels between samples were performed with the R package limma [108,109] on log_2_ normalized intensity data exported from Scaffold. The Benjamini-Hochberg method was used to adjust the P-value. Proteins with adjusted P-value < 0.015 were considered to have significant changes in abundance between samples, regardless of magnitude of log_2_ fold change.

#### Gene ontology analysis

Gene ontology analyses of proteins enriched (“Up”) or depleted (“Down”) or mRNAs with increased (“Up”) or decreased (“Down”) TE were carried out by the R package gprofiler2’s gost function with default parameters [110,111].

#### mRNA features

mRNA features related to analysis of stop codon readthrough efficiency were defined as previously described [84].

Identification of uORFs in the 5’-UTR was done with the findORFs function in the R package ORFik [112], limiting uORF’s start codon to be AUG only and no minimum uORF length.

Poly(A) tracts in the 5’-UTR and 3’-UTR were defined as stretches of at least 10 consecutive adenines (the A in AUG of main ORF included), allowing at most 2 other nucleotides in the 10 A’s window.

Oligo(U) in the 5’-UTR and 3’-UTR was defined as a stretch of at least 7 consecutive uracils, allowing no other nucleotides in the window.

Codon optimality score for each transcript’s CDS was calculated as described previously [84,93]. For a given codon, a codon optimality measurement was defined as the tRNA adaptation index (tAI) derived from tRNA gene copy numbers and wobble base-pairing penalty [113,114]. The geometric mean of tAIs of all codons in a given CDS was the codon optimality score for that CDS [113].

#### Motif discovery

Identification of sequence motifs enriched in 5’-UTR (excluding the start codon) or 3’-UTR (including the stop codon) sequences of mRNA TE groups compared to the Reference was carried out by the STREME algorithm from the MEME Suite 5.5.0 (https://meme-suite.org/meme/tools/streme) [76,115]. A general search for 3–15 nt-long motifs was performed as well as a focused search for shorter 3–6 nt-long motifs. Short motifs had to be identified in both searches to be regarded as significant enrichments.

#### Statistical analyses

Statistical analyses were performed using the R packages rstatix, rcompanion, and ggpubr. Specific statistical parameters, multiple-testing correction method, statistical significance (p-value or symbols representing ranges of p-values), and sample size (n) are reported accordingly on the figures, in the figure legends, or in S3 Table.

## Supporting information

S1 Fig Replicate reproducibilities. **A.** Correlation matrix showing Pearson correlation coefficients (*r*) of log_2_ intensity values from mass spectrometry data between pairs of samples. **B.** Correlation matrix showing Pearson correlation coefficients (*r*) of transcript abundance (non-zero RPKM values, log_10_-transformed) between pairs of sequencing libraries, RNA-seq (RNA) and ribosome profiling (RPF = ribosome-protected fragment) libraries.
(TIF)

S2 Fig Changes in transcriptomes and proteomes of *pbp1Δ* and *pab1Δpbp1Δ* strains relative to WT. **A.** Volcano plots of changes in proteome (mass spectrometry data) between strains. Orange, purple, and grey dots represent proteins with higher abundance (positive log_2_ fold change, adjusted p-value < 0.015), lower abundance (negative log_2_ fold change, adjusted p-value < 0.015), and no change (adjusted p-value ≥ 0.015), respectively. **B.** Volcano plots of changes in transcriptome (RNA-Seq data) between strains. Orange, purple, and grey dots represent mRNAs with higher abundance (positive log_2_ fold change, adjusted p-value < 0.01), lower abundance (negative log_2_ fold change, adjusted p-value < 0.01), and no change (adjusted p-value ≥ 0.01), respectively. **C.** Comparison of log_2_ fold change in transcriptome (RNA-Seq reads) and proteome (mass spectrometry quantification), with Spearman’s correlation coefficient. **D.** Comparison of log_2_ fold change in ribosome profiling (Ribo-Seq) reads and proteome (mass spectrometry quantification), with Spearman’s correlation coefficient. For C and D: Grey, genes whose mRNA and protein abundance remained unchanged. Blue, genes whose protein but not mRNA abundance changed significantly. Red, genes whose mRNA but not protein abundance changed significantly. Green, genes whose mRNA and protein abundance both changed significantly.
(TIF)

S3 Fig Gene ontology analysis of protein abundance changes in *pbp1Δ* relative *WT*. Manhattan plot was generated by the gostplot function in the R package gprofiler2 [111]. Each circle on the plot represents a gene ontology (GO) term. The size of the circle reflects the number of genes in the GO term. GO terms are grouped and colored by data sources on the x-axis. GO terms that are closer in hierarchy are also closer visually along the x-axis. The y-axis shows adjusted p-values in negative log_10_ scale. The plot is capped at 16, meaning GO terms with adjusted p-value < 10^−16^ are plotted at “>16” on the y-axis. All GO terms in each category (e.g., BP: Biological Process, MF: Molecular Function, etc.) are labeled and provided as a table below the plot.
(TIF)

S4 Fig Gene ontology analysis of protein abundance changes in *pab1Δpbp1Δ* relative *pbp1Δ*. Manhattan plot was generated by the gostplot function in the R package gprofiler2 [111]. Each circle on the plot represents a gene ontology (GO) term. The size of the circle reflects the number of genes in the GO term. GO terms are grouped and colored by data sources on the x-axis. GO terms that are closer in hierarchy are also closer visually along the x-axis. The y-axis shows adjusted p-values in negative log_10_ scale. The plot is capped at 16, meaning GO terms with adjusted p-value < 10^−16^ are plotted at “>16” on the y-axis. Top 3 GO terms in each category (e.g., BP: Biological Process, MF: Molecular Function, etc.) based on the adjusted p-value are labeled and provided as a table below the plot.
(TIF)

S5 Fig Changes in RNA-Seq reads, Ribo-Seq reads, and proteomics data in *pab1Δpbp1Δ* relative *pbp1Δ*. **A.** As in Fig 1C, with genes partitioned into whether relative translation efficiency (TE) significantly changes. **B.** As in Fig 1D, with genes partitioned into whether relative translation efficiency (TE) significantly changes.
(TIF)

S6 Fig Gene ontology analysis of translation efficiency changes in *pab1Δpbp1Δ* relative *pbp1Δ*. Manhattan plot was generated by the gostplot function in the R package gprofiler2 [111]. Each circle on the plot represents a gene ontology (GO) term. The size of the circle reflects the number of genes in the GO term. GO terms are grouped and colored by data sources on the x-axis. GO terms that are closer in hierarchy are also closer visually along the x-axis. The y-axis shows adjusted p-values in negative log_10_ scale. The plot is capped at 16, meaning GO terms with adjusted p-value < 10^−16^ are plotted at “>16” on the y-axis. Top 3 GO terms in each category (e.g., BP: Biological Process, MF: Molecular Function, etc.) based on the adjusted p-value are labeled and provided as a table below the plot.
(TIF)

S7 Fig Influences of translation initiation features on translation efficiency (TE) changes. **A.** Comparison of log_2_ fold change in TE upon *PAB1* deletion and log_2_ fold enrichment in eIF4G or Pab1 RIP-seq [36], with Spearman’s correlation coefficient. **B.** Cumulative density plots of log_2_ fold change in TE upon *PAB1* deletion of mRNAs with (“Yes”) or without (“No”) uORF. Two-sided Kolmogorov-Smirnov (KS) test was used to determine significant difference between groups. **C.** Influences of start codon context on TE changes. Relative proportions of nucleotide usage upstream (**top**) and downstream (**middle**) of main ORF’s AUG (positions +1 +2 +3) in Up and Down groups relative to Reference. Relative proportions of nucleotide usage from the mRNA 5’ cap (first 18 nucleotides of the 5’-UTR sequences) in Up and Down groups relative to Reference (**bottom**). In all panels, analyses were limited to mRNAs with existing UTR annotations. Reference (Ref.) group includes all mRNAs regardless of TE changes (Up + Down + Unchanged) to recapitulate the general proportions in the transcriptome. Positive (red) and negative (blue) log_2_ relative proportion indicates that the nucleotide is over-represented and under-represented, respectively, in the group compared to the Reference. Pairwise χ^2^ test with Benjamini-Hochberg method for multiple-testing correction was used to compare the nucleotide frequencies between Reference, Up, and Down groups. p < 0.05 for Up or Down vs. Reference is represented by a big tile, while non-significant results are represented by a small tile. p < 0.05 for Up vs. Down is represented by an asterisk (“*”).
(TIF)

S8 Fig Relationships between closed-loop component association and transcript length with regard to translation efficiency (TE). Distribution of mRNA transcript lengths grouped by TE changes from Fig 5A and enrichment or depletion in eIF4G or Pab1 (RIP-seq experiments), comparing by RIP-seq status. Two-sided Wilcoxon’s rank sum test with Benjamini-Hochberg method for multiple-testing correction was used to compare values between pairwise groups. Only significant comparisons were reported as the following: (*) p < 0.05, (**) p < 0.01, (***) p < 0.001, (****) p < 0.0001. Analyses were limited to mRNAs with existing UTR annotations. Reference (Ref.) group includes all mRNAs regardless of TE changes (Up + Down + Unchanged) to recapitulate the general distribution of measured values in the transcriptome.
(TIF)

S9 Fig Out-of-frame translation in ribosome profiling data. **A.** Comparison of 3’-UTR footprint density (RPKM) and fraction of out-of-frame footprints in the last 30 nt (10 codon) of the CDS, with Spearman’s correlation coefficient. mRNAs were required to have UTR annotations, RPKM of the CDS > 0.2, at least 30 footprints across the last 30 nt of CDS, and RPKM of the 3’-UTR > 0.1 to be included in the analysis. **B.** Distribution of 3’-UTR footprint density (RPKM) of mRNAs with (“Yes”) or without (“No”) out-of-frame stop codon(s) within the CDS region. Two-sided Wilcoxon’s rank sum test with Benjamini-Hochberg was used to compare values between groups. mRNAs were required to have UTR annotations, RPKM of the CDS > 0.2, and RPKM of the 3’-UTR > 0.1 to be included in the analysis.
(TIF)

S10 Fig Influences of mRNA features on readthrough efficiency. **A.** Average ± standard deviation of performance metrics (normalized root mean squared error (NRMSE)) extracted from 25 random forest models (5-fold cross-validation, repeated 5 times) trained for each strain to predict readthrough efficiency. **B**-**C**. Heatmaps of median readthrough efficiency of mRNA groups, grouped by the identity of the stop codon or the identity of nucleotide at positions near the stop codon (**B**) or the identity of P-site codon (**C**), relative to median readthrough efficiency of all mRNAs in the sample. Positive (red) and negative (blue) values indicate that the group has higher and lower readthrough efficiency than the sample median, respectively. Two-sided Wilcoxon’s rank sum test with Benjamini-Hochberg method for multiple-testing correction was used to compare a group’s median readthrough efficiency to the sample median. Significant results (p < 0.05) are represented as bigger tiles.
(TIF)

S1 Table Gene ontology analysis results of proteins with significant changes in abundance in *pab1Δpbp1Δ* vs. *pbp1Δ* and *pbp1Δ* vs. WT. (XLSX)

S2 Table Gene ontology analysis results of mRNAs with significant changes in translation efficiency (TE) in *pab1Δpbp1Δ* vs. *pbp1Δ*. (XLSX)

S3 Table Statistical test results of pairwise comparison of mRNA features between Reference, Up, and Down TE groups. Related to Figs 5F, 6B, 6C, 6D, 7D and 7F.
(XLSX)

S4 Table List of yeast strains and genotypes used in this study. (XLSX)

S5 Table List of oligonucleotides used in this study. (XLSX)

S6 Table Ribosome profiling reads processing and alignment statistics. (XLSX)

S7 Table Ribosome footprint P-site offsets from footprint’s 5’ end and 3’ ends. (XLSX)
