# Supplementary figures and images for: Pleiotropic effects of PAB1 deletion: Extensive changes in the yeast proteome, transcriptome, and translatome
Source: PLoS Genet. 2024 Sep 5;20(9):e1011392. doi: 10.1371/journal.pgen.1011392 (PMC11407637; doi:10.1371/journal.pgen.1011392)

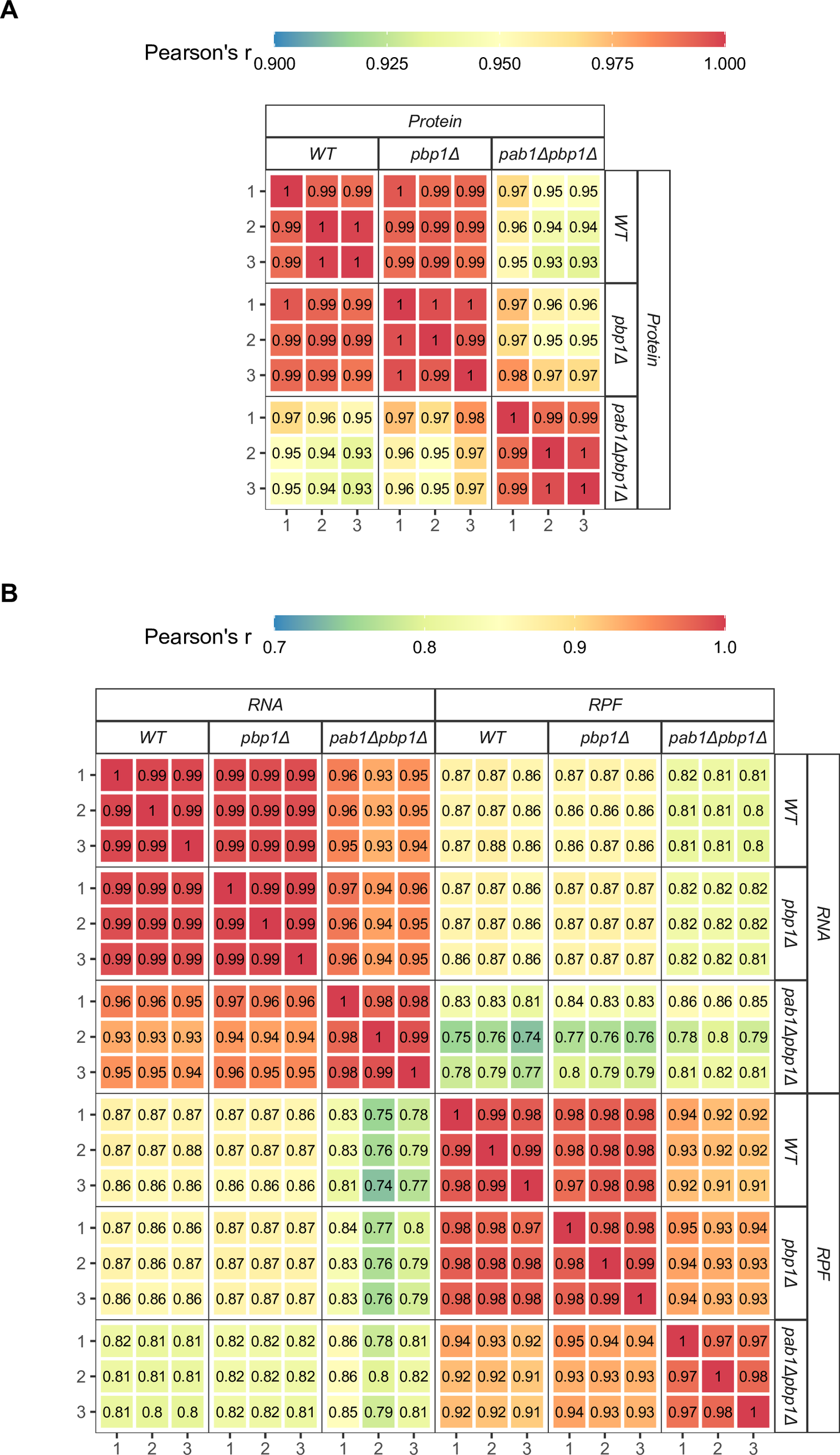

Supplement: S1 Fig — A. Correlation matrix showing Pearson correlation coefficients (r) of log2 intensity values from mass spectrometry data between pairs of samples. B. Correlation matrix showing Pearson correlation coefficients (r) of transcript abundance (non-zero RPKM values, log10-transformed) between pairs of sequencing libraries, RNA-seq (RNA) and ribosome profiling (RPF = ribosome-protected fragment) libraries. (TIF) [file pgen.1011392.s001.tif]

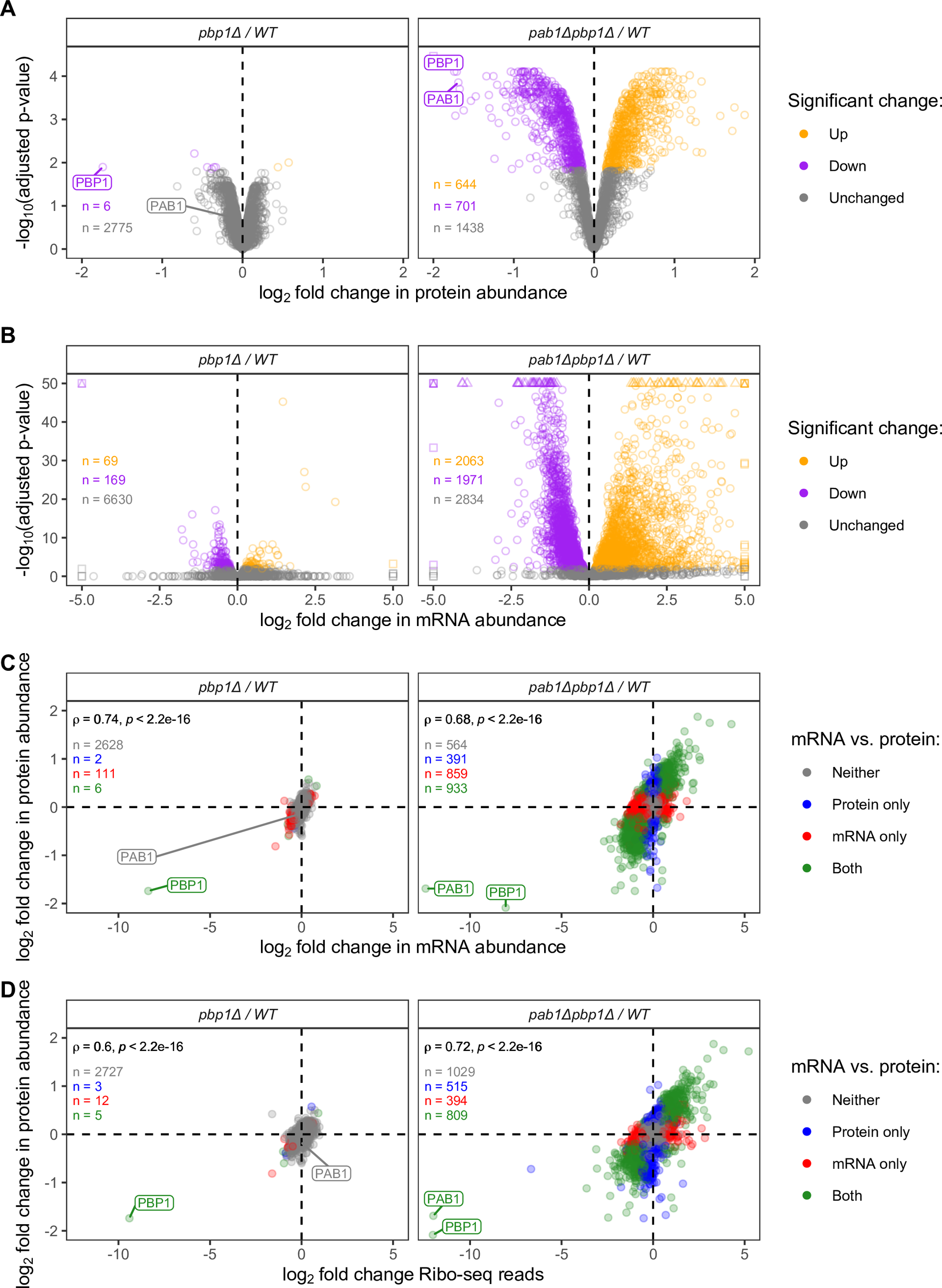

Supplement: S2 Fig — A. Volcano plots of changes in proteome (mass spectrometry data) between strains. Orange, purple, and grey dots represent proteins with higher abundance (positive log2 fold change, adjusted p-value < 0.015), lower abundance (negative log2 fold change, adjusted p-value < 0.015), and no change (adjusted p-value ≥ 0.015), respectively. B. Volcano plots of changes in transcriptome (RNA-Seq data) between strains. Orange, purple, and grey dots represent mRNAs with higher abundance (positive log2 fold change, adjusted p-value < 0.01), lower abundance (negative log2 fold change, adjusted p-value < 0.01), and no change (adjusted p-value ≥ 0.01), respectively. C. Comparison of log2 fold change in transcriptome (RNA-Seq reads) and proteome (mass spectrometry quantification), with Spearman’s correlation coefficient. D. Comparison of log2 fold change in ribosome profiling (Ribo-Seq) reads and proteome (mass spectrometry quantification), with Spearman’s correlation coefficient. For C and D: Grey, genes whose mRNA and protein abundance remained unchanged. Blue, genes whose protein but not mRNA abundance changed significantly. Red, genes whose mRNA but not protein abundance changed significantly. Green, genes whose mRNA and protein abundance both changed significantly. (TIF) [file pgen.1011392.s002.tif]

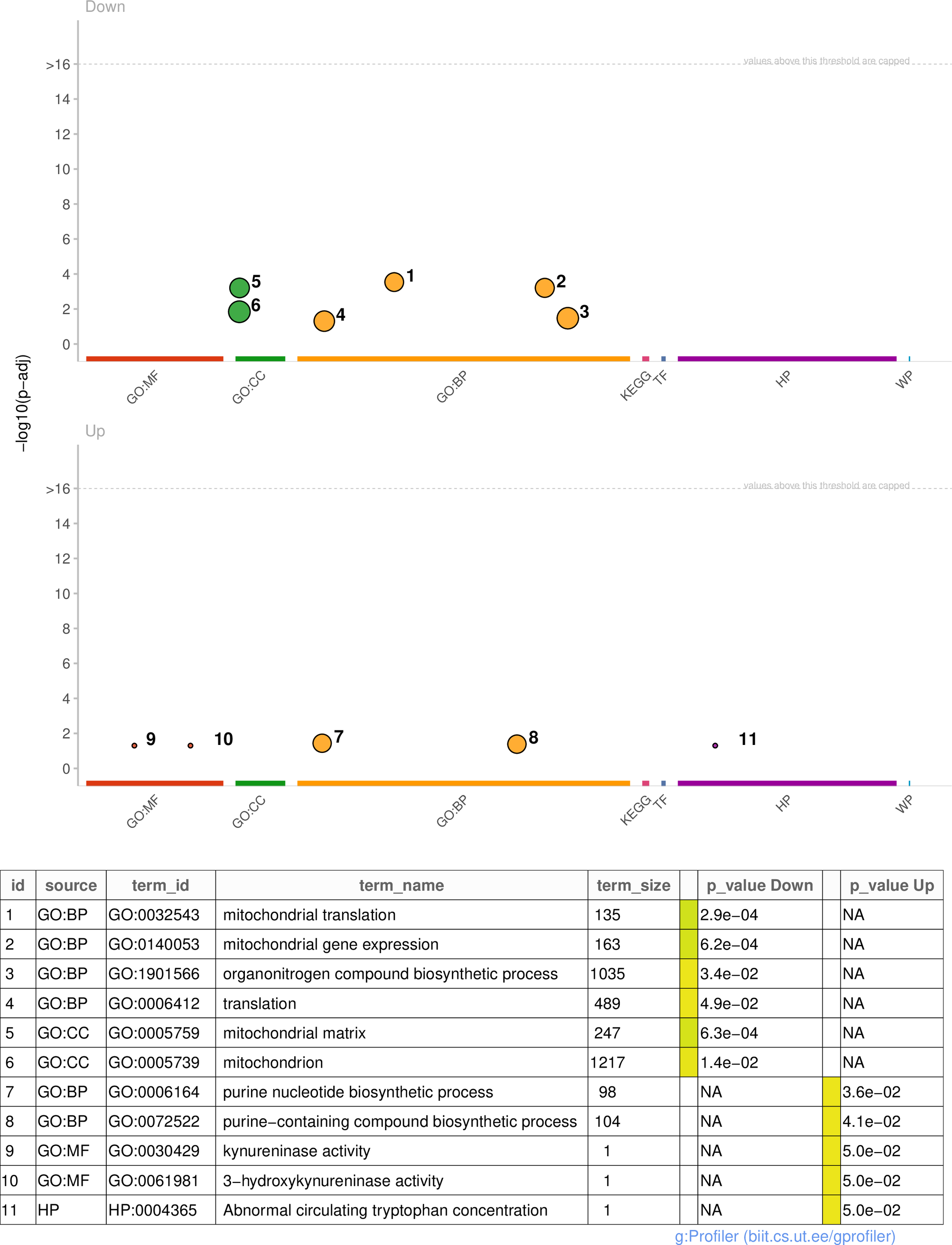

Supplement: S3 Fig — Manhattan plot was generated by the gostplot function in the R package gprofiler2 [111]. Each circle on the plot represents a gene ontology (GO) term. The size of the circle reflects the number of genes in the GO term. GO terms are grouped and colored by data sources on the x-axis. GO terms that are closer in hierarchy are also closer visually along the x-axis. The y-axis shows adjusted p-values in negative log10 scale. The plot is capped at 16, meaning GO terms with adjusted p-value < 10−16 are plotted at “>16” on the y-axis. All GO terms in each category (e.g., BP: Biological Process, MF: Molecular Function, etc.) are labeled and provided as a table below the plot. (TIF) [file pgen.1011392.s003.tif]

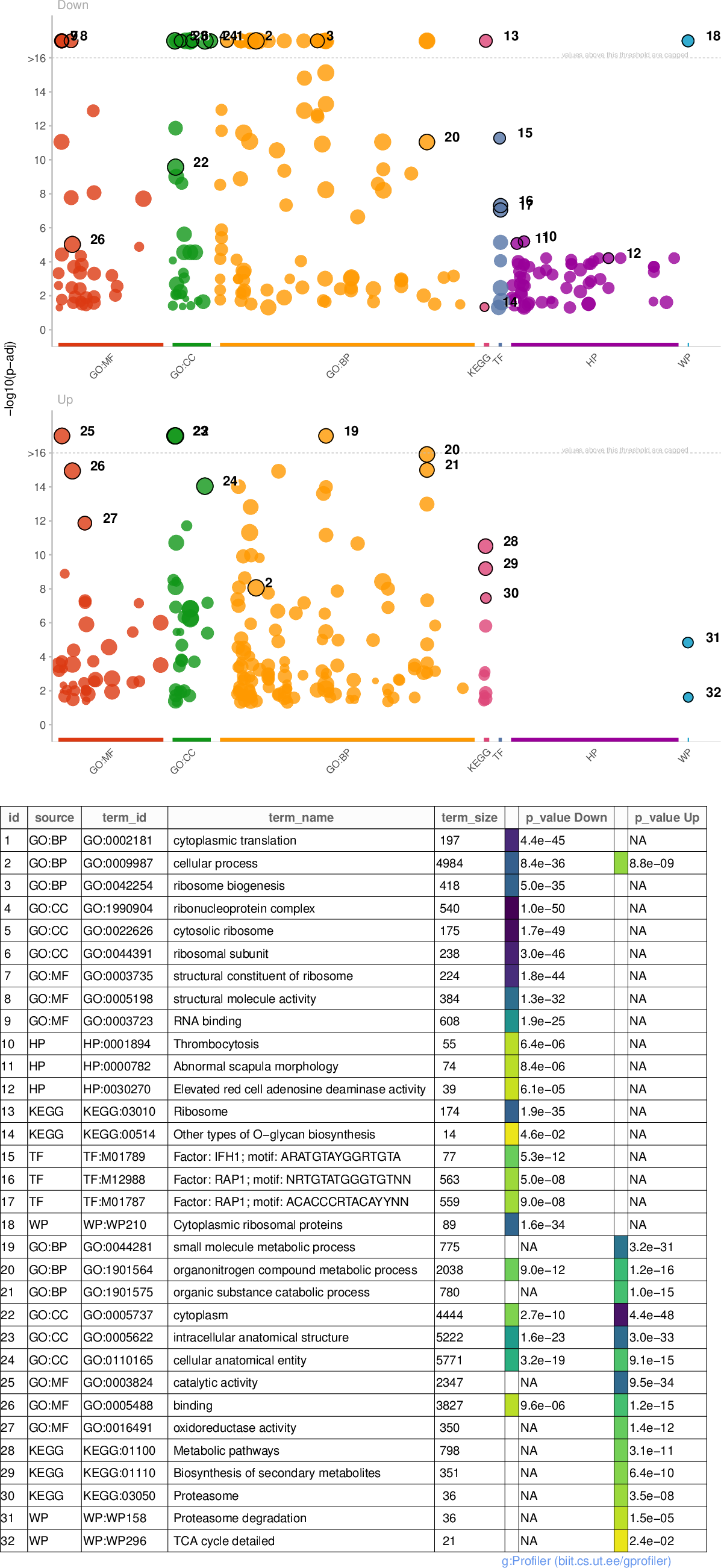

Supplement: S4 Fig — Manhattan plot was generated by the gostplot function in the R package gprofiler2 [111]. Each circle on the plot represents a gene ontology (GO) term. The size of the circle reflects the number of genes in the GO term. GO terms are grouped and colored by data sources on the x-axis. GO terms that are closer in hierarchy are also closer visually along the x-axis. The y-axis shows adjusted p-values in negative log10 scale. The plot is capped at 16, meaning GO terms with adjusted p-value < 10−16 are plotted at “>16” on the y-axis. Top 3 GO terms in each category (e.g., BP: Biological Process, MF: Molecular Function, etc.) based on the adjusted p-value are labeled and provided as a table below the plot. (TIF) [file pgen.1011392.s004.tif]

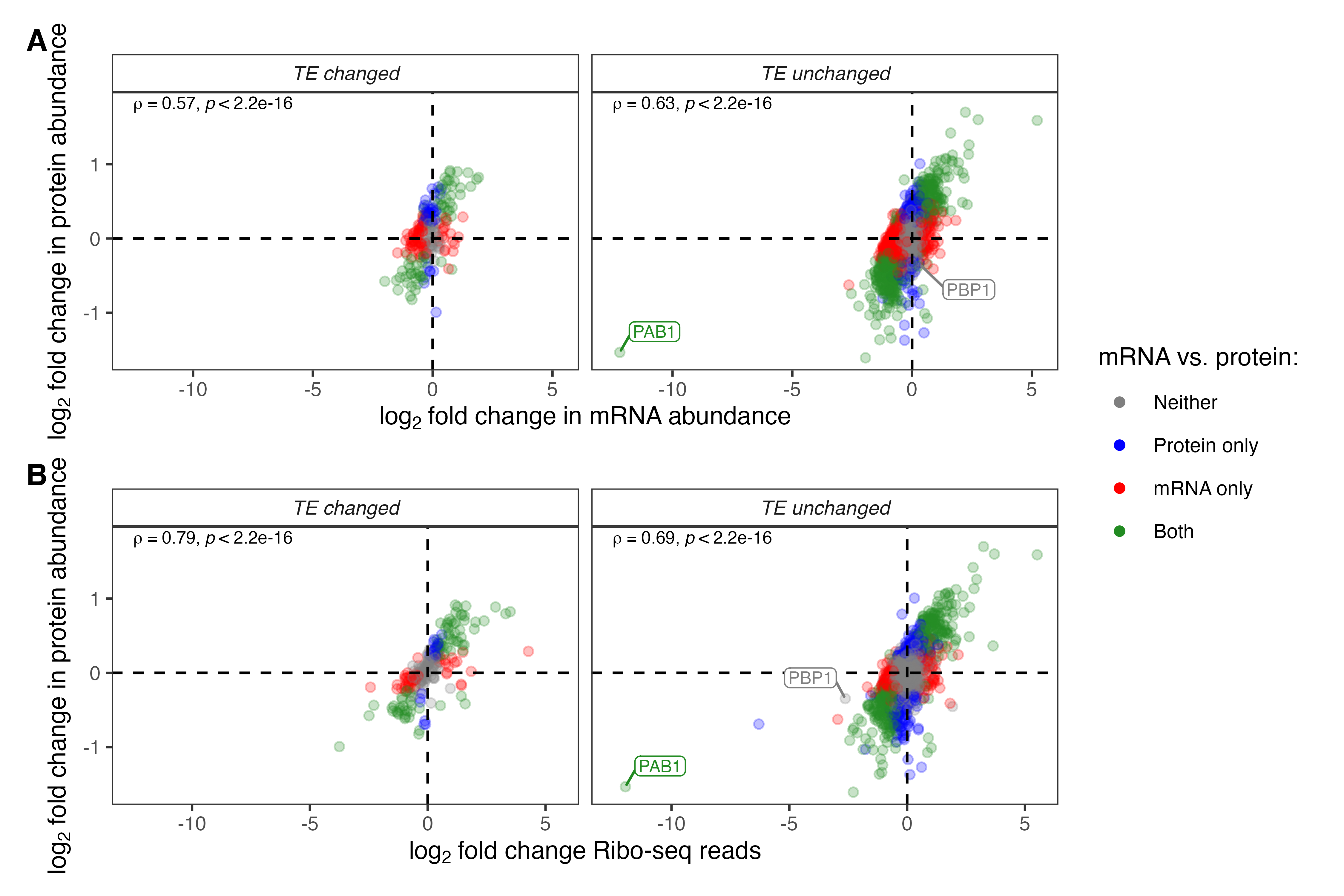

Supplement: S5 Fig — A. As in Fig 1C, with genes partitioned into whether relative translation efficiency (TE) significantly changes. B. As in Fig 1D, with genes partitioned into whether relative translation efficiency (TE) significantly changes. (TIF) [file pgen.1011392.s005.tif]

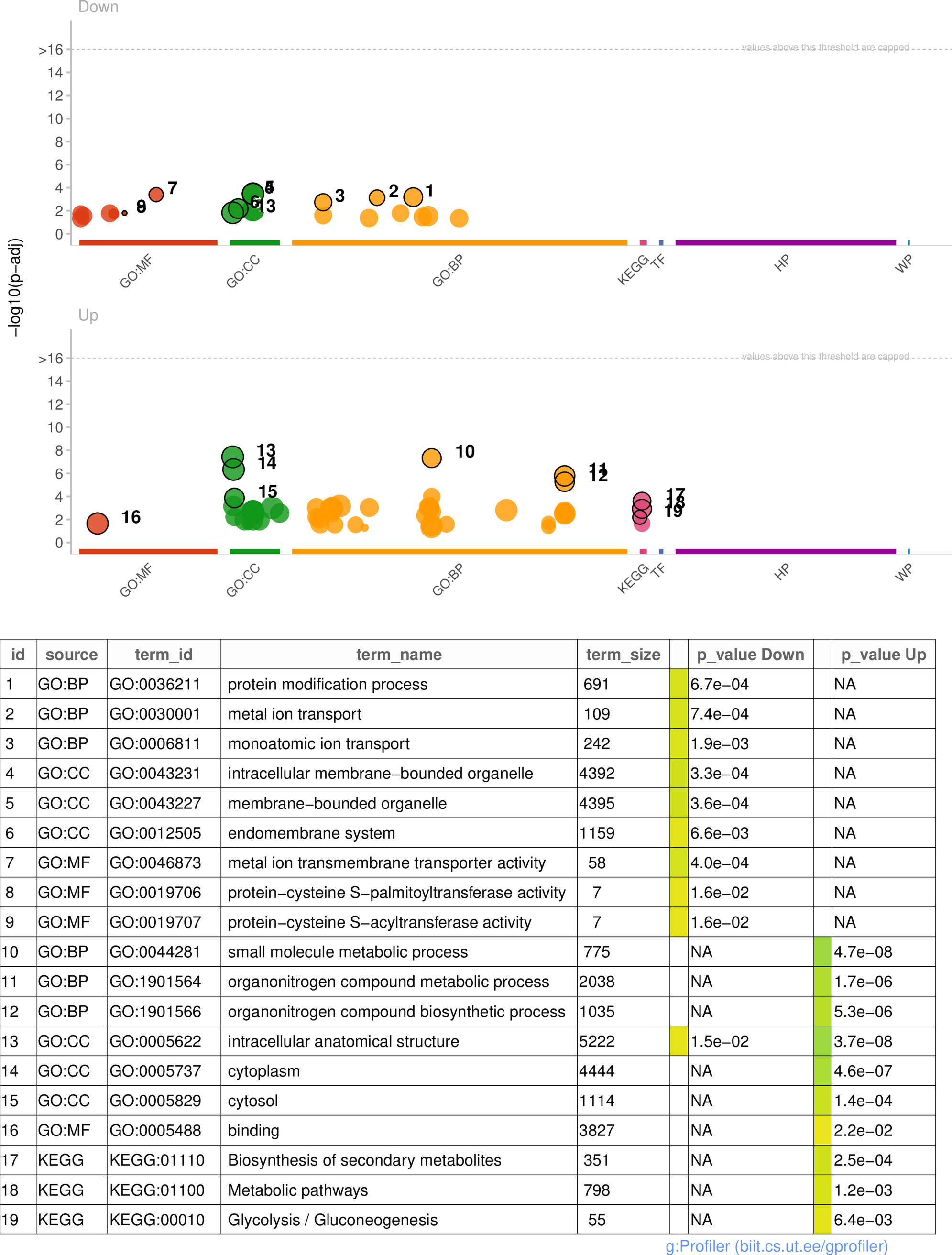

Supplement: S6 Fig — Manhattan plot was generated by the gostplot function in the R package gprofiler2 [111]. Each circle on the plot represents a gene ontology (GO) term. The size of the circle reflects the number of genes in the GO term. GO terms are grouped and colored by data sources on the x-axis. GO terms that are closer in hierarchy are also closer visually along the x-axis. The y-axis shows adjusted p-values in negative log10 scale. The plot is capped at 16, meaning GO terms with adjusted p-value < 10−16 are plotted at “>16” on the y-axis. Top 3 GO terms in each category (e.g., BP: Biological Process, MF: Molecular Function, etc.) based on the adjusted p-value are labeled and provided as a table below the plot. (TIF) [file pgen.1011392.s006.tif]

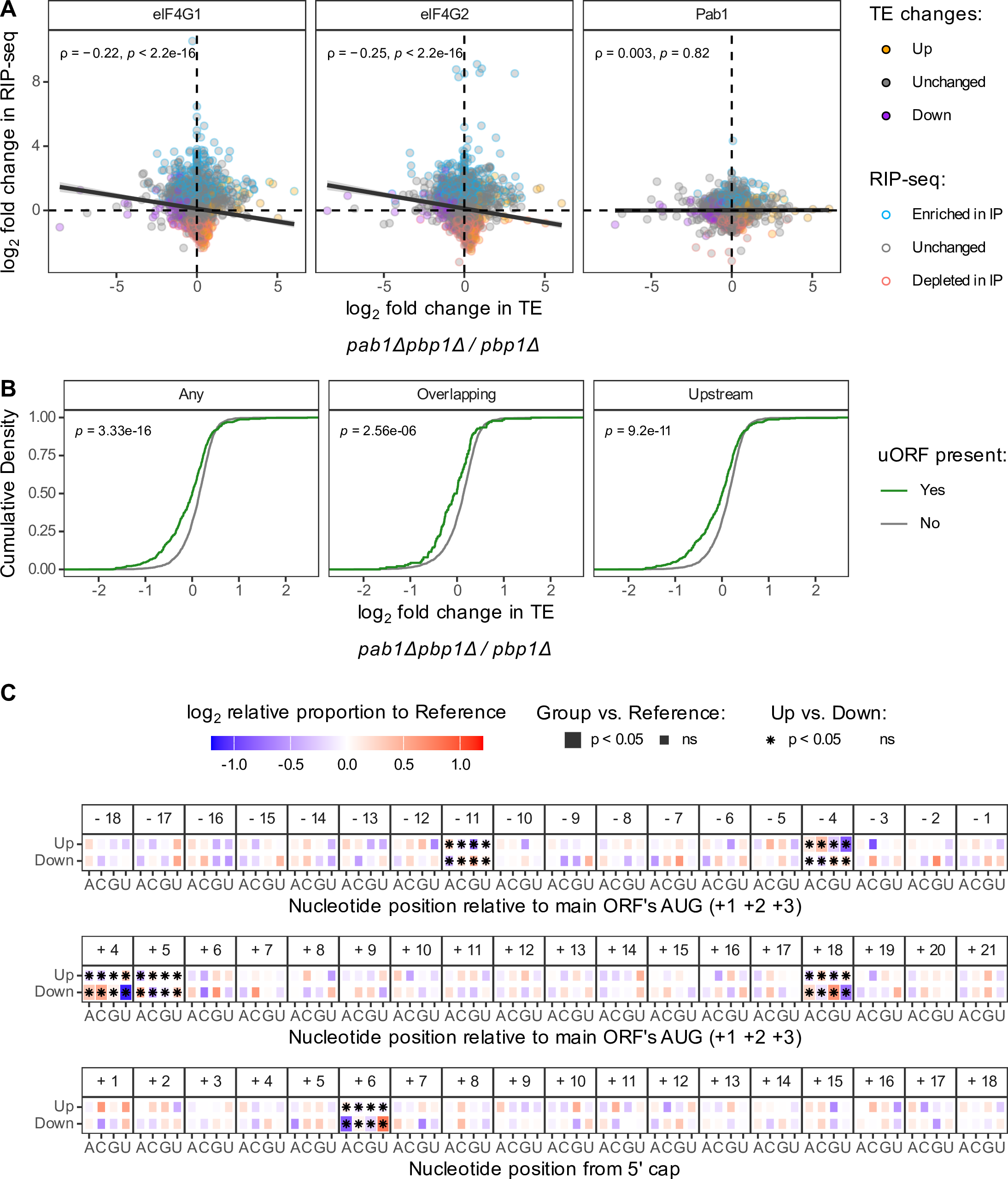

Supplement: S7 Fig — A. Comparison of log2 fold change in TE upon PAB1 deletion and log2 fold enrichment in eIF4G or Pab1 RIP-seq [36], with Spearman’s correlation coefficient. B. Cumulative density plots of log2 fold change in TE upon PAB1 deletion of mRNAs with (“Yes”) or without (“No”) uORF. Two-sided Kolmogorov-Smirnov (KS) test was used to determine significant difference between groups. C. Influences of start codon context on TE changes. Relative proportions of nucleotide usage upstream (top) and downstream (middle) of main ORF’s AUG (positions +1 +2 +3) in Up and Down groups relative to Reference. Relative proportions of nucleotide usage from the mRNA 5’ cap (first 18 nucleotides of the 5’-UTR sequences) in Up and Down groups relative to Reference (bottom). In all panels, analyses were limited to mRNAs with existing UTR annotations. Reference (Ref.) group includes all mRNAs regardless of TE changes (Up + Down + Unchanged) to recapitulate the general proportions in the transcriptome. Positive (red) and negative (blue) log2 relative proportion indicates that the nucleotide is over-represented and under-represented, respectively, in the group compared to the Reference. Pairwise χ2 test with Benjamini-Hochberg method for multiple-testing correction was used to compare the nucleotide frequencies between Reference, Up, and Down groups. p < 0.05 for Up or Down vs. Reference is represented by a big tile, while non-significant results are represented by a small tile. p < 0.05 for Up vs. Down is represented by an asterisk (“*”). (TIF) [file pgen.1011392.s007.tif]

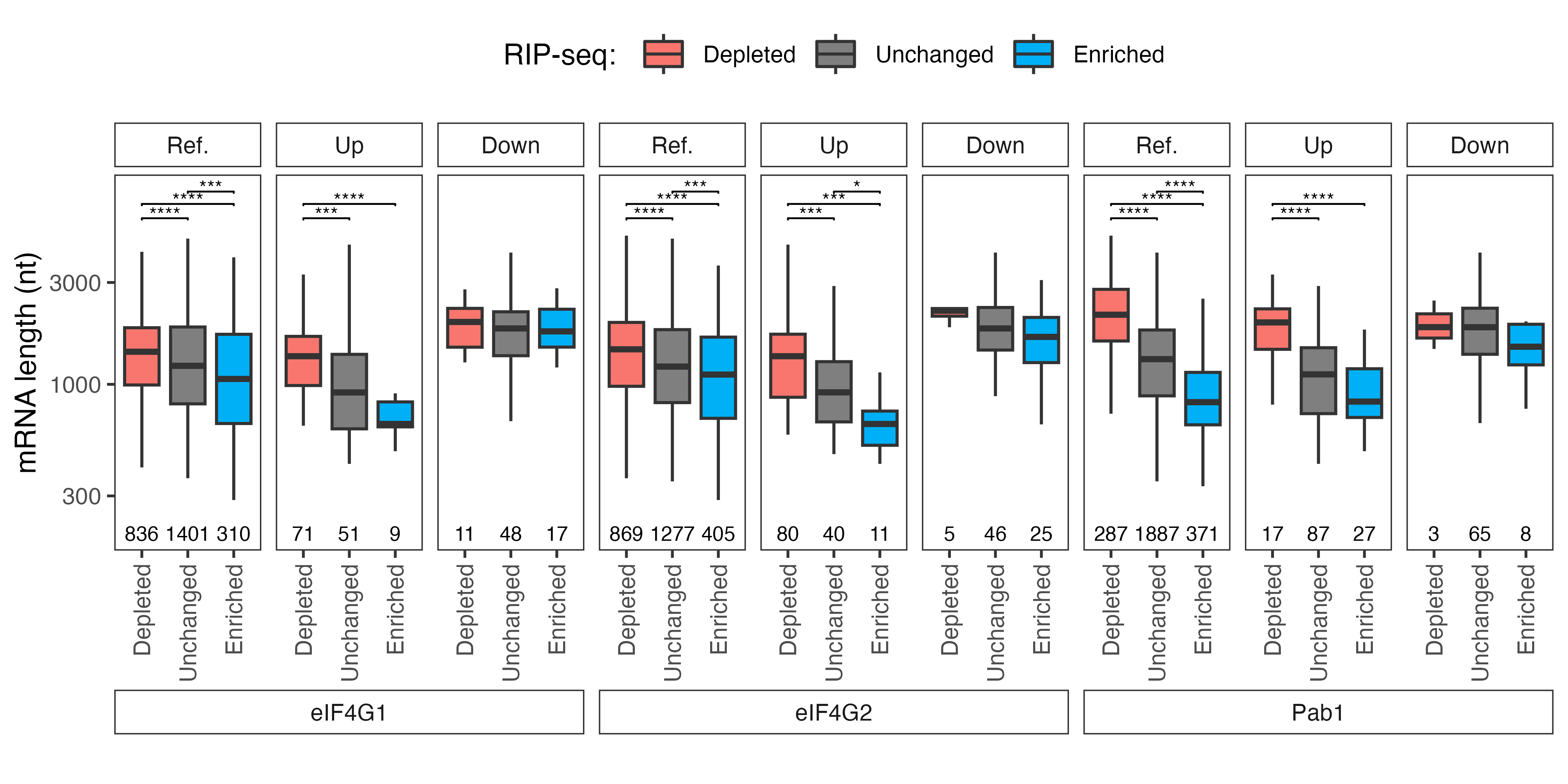

Supplement: S8 Fig — Distribution of mRNA transcript lengths grouped by TE changes from Fig 5A and enrichment or depletion in eIF4G or Pab1 (RIP-seq experiments), comparing by RIP-seq status. Two-sided Wilcoxon’s rank sum test with Benjamini-Hochberg method for multiple-testing correction was used to compare values between pairwise groups. Only significant comparisons were reported as the following: (*) p < 0.05, (**) p < 0.01, (***) p < 0.001, (****) p < 0.0001. Analyses were limited to mRNAs with existing UTR annotations. Reference (Ref.) group includes all mRNAs regardless of TE changes (Up + Down + Unchanged) to recapitulate the general distribution of measured values in the transcriptome. (TIF) [file pgen.1011392.s008.tif]

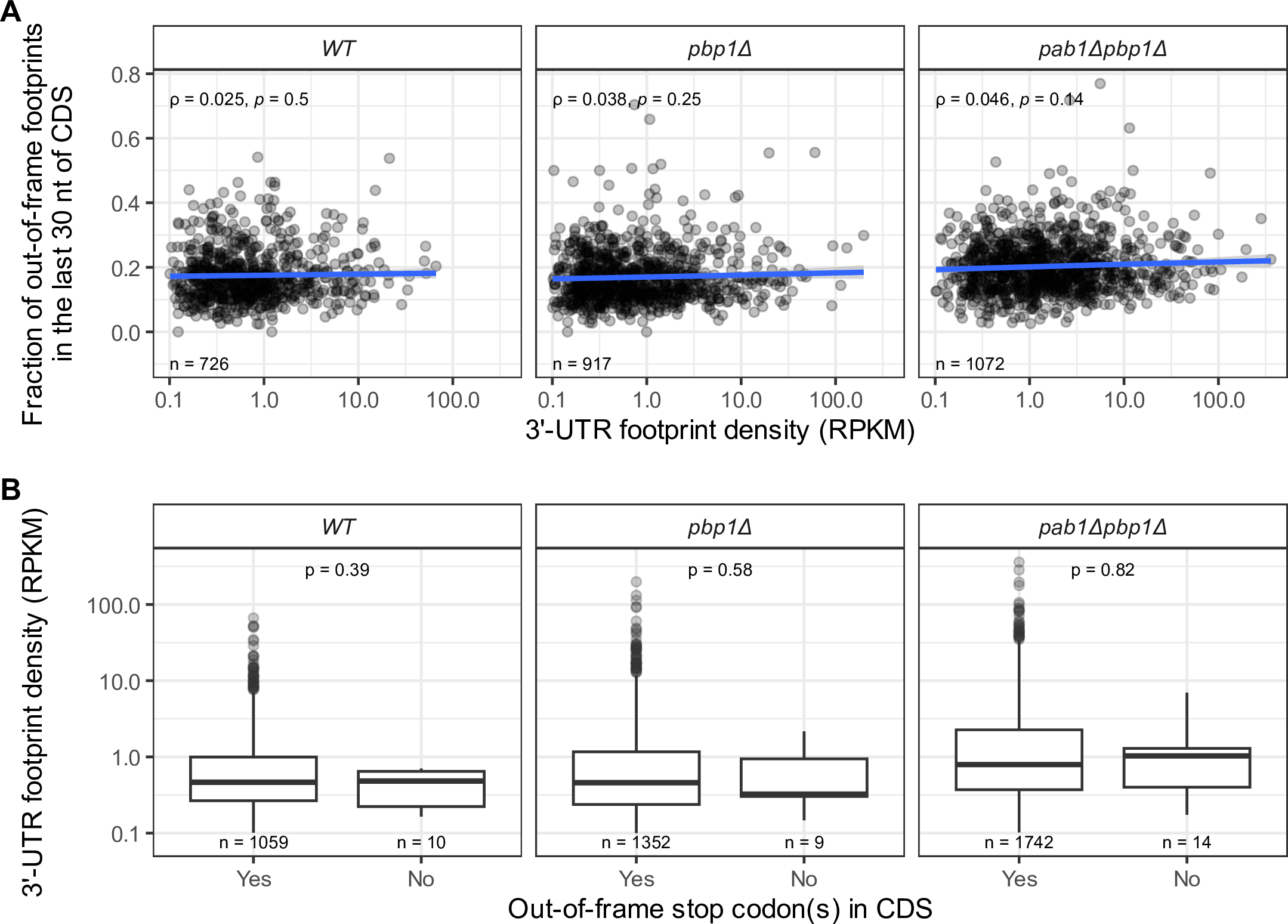

Supplement: S9 Fig — A. Comparison of 3’-UTR footprint density (RPKM) and fraction of out-of-frame footprints in the last 30 nt (10 codon) of the CDS, with Spearman’s correlation coefficient. mRNAs were required to have UTR annotations, RPKM of the CDS > 0.2, at least 30 footprints across the last 30 nt of CDS, and RPKM of the 3’-UTR > 0.1 to be included in the analysis. B. Distribution of 3’-UTR footprint density (RPKM) of mRNAs with (“Yes”) or without (“No”) out-of-frame stop codon(s) within the CDS region. Two-sided Wilcoxon’s rank sum test with Benjamini-Hochberg was used to compare values between groups. mRNAs were required to have UTR annotations, RPKM of the CDS > 0.2, and RPKM of the 3’-UTR > 0.1 to be included in the analysis. (TIF) [file pgen.1011392.s009.tif]

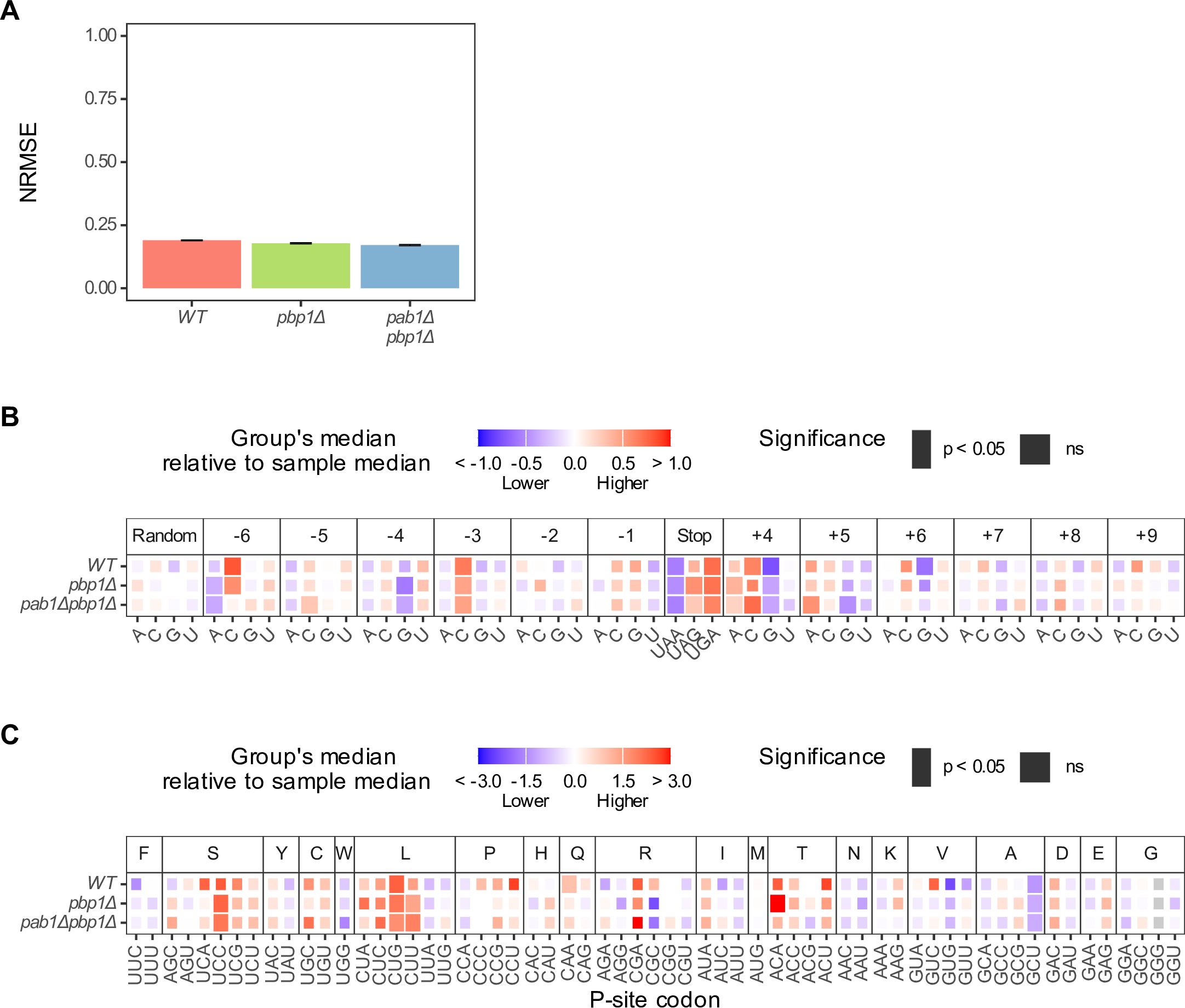

Supplement: S10 Fig — A. Average ± standard deviation of performance metrics (normalized root mean squared error (NRMSE)) extracted from 25 random forest models (5-fold cross-validation, repeated 5 times) trained for each strain to predict readthrough efficiency. B-C. Heatmaps of median readthrough efficiency of mRNA groups, grouped by the identity of the stop codon or the identity of nucleotide at positions near the stop codon (B) or the identity of P-site codon (C), relative to median readthrough efficiency of all mRNAs in the sample. Positive (red) and negative (blue) values indicate that the group has higher and lower readthrough efficiency than the sample median, respectively. Two-sided Wilcoxon’s rank sum test with Benjamini-Hochberg method for multiple-testing correction was used to compare a group’s median readthrough efficiency to the sample median. Significant results (p < 0.05) are represented as bigger tiles. (TIF) [file pgen.1011392.s010.tif]
